# Service learning for improving academic success in students in grade K to 12: A systematic review

**DOI:** 10.1002/cl2.1210

**Published:** 2022-01-07

**Authors:** Trine Filges, Jens Dietrichson, Bjørn C. A. Viinholt, Nina T. Dalgaard

**Affiliations:** ^1^ VIVE—The Danish Center for Social Science Research Copenhagen Denmark

## Abstract

**Background:**

School‐based service‐learning is a teaching strategy that explicitly links community service to academic instruction. It is distinctive from traditional voluntarism or community service in that it intentionally connects service activities with curriculum concepts and includes structured time for reflection. Service learning, by connecting education to real world issues and allowing students to address problems they identify, may be particularly efficacious as it increases engagement and motivates students, in particular students who might not respond well to more traditional teaching methods.

**Objectives:**

The main objective was to answer the following research question: What are the effects of service learning on academic success, neither employed, nor in education or training (NEET) status post compulsory school, personal and social skills, and risk behaviour of students in primary and secondary education (grades kindergarten to 12)? Further, we wanted to investigate study‐level summaries of participant characteristics (e.g., gender, age or socioeconomic level) and quality of the service learning programme.

**Search Methods:**

We identified relevant studies through electronic searches of bibliographic databases, governmental and grey literature repositories, hand search in specific targeted journals, citation tracking, and Internet search engines. The database searches were carried out in November 2019 and other resources were searched in October 2020. We searched to identify both published and unpublished literature, and reference lists of included studies and relevant reviews were searched.

**Selection Criteria:**

The intervention was service learning which can be described as a curriculum‐based community service that integrates classroom instruction (such as classroom discussions, presentations, or directed writing) with community service activities. We included children in primary and secondary education (grades kindergarten to 12) in general education. Our primary focus was on measures of academic success and NEET status. A secondary focus was on measures of personal and social skills, and risk behaviour (such as drug and alcohol use, violent behaviour, sexual risk taking). All study designs that used a well‐defined control group were eligible for inclusion. Studies that utilised qualitative approaches were not included.

**Data Collection and Analysis:**

The total number of potentially relevant studies constituted 13,719 hits. A total of 37 studies met the inclusion criteria. The 37 studies analysed 30 different populations. Only 10 studies (analysing nine different populations) could be used in the data synthesis. Eighteen studies could not be used in the data synthesis as they were judged to have critical risk of bias and, in accordance with the protocol, were excluded from the meta‐analysis on the basis that they would be more likely to mislead than inform. Five studies did not provide enough information enabling us to calculate an effects size and standard error, and one study did not provide enough information to assess risk of bias. Finally, two clusters of studies used the same data sets, resulting in an additional three studies we did not use in the data synthesis. Meta‐analysis of all outcomes were conducted on each conceptual outcome separately. All analyses were inverse variance weighted using random effects statistical models incorporating both the sampling variance and between study variance components into the study level weights. Random effects weighted mean effect sizes were calculated using 95% confidence intervals. We carried out a sensitivity analysis to examine the impact of correcting for clustered assignment of treatments.

**Main Results:**

The 10 studies (analysing nine different populations) used for meta analysis were all from the United States. The timespan in which included studies were carried out was 33 years, from 1980 to 2013; on average the intervention year was 2007. The average number of participants in the analysed service learning interventions was 937, ranging from 18 to 3556 and the average number of controls was 927, ranging from 20 to 3395. At most, the results from three studies could be pooled in any of the meta‐analyses. All the meta‐analyses showed a weighted average that favoured the intervention group except the pregnancy outcome. None of them was statistically significant except the weighted average of the two studies reporting math test results. The random effects weighted standardised mean difference was 0.09 [95% confidence interval (CI): −0.02 to 0.21] for students' general grade point average; 0.04 (95% CI: −0.08 to 0.16) for reading; 0.21 (95% CI: 0.09 to 0.33) for math; 0.03 (95% CI: −0.10 to 0.16) for days absent from school; 0.13 (95% CI: −0.14 to 0.40) for self‐esteem; 0.07 (95% CI: −0.04 to 0.18) for locus of control. The random effects weighted odds ratio was 1.05 (95% CI: 0.63 to 1.74) for pregnancy and 0.96 (95% CI: 0.74 to 1.25) for sexual risk behaviour. In addition, a number of other outcomes were reported in a single study only. There were no appreciable changes in the results as indicated by the sensitivity analysis. We did not find any adverse effects.

**Authors' Conclusions:**

In this review, we aimed to find evidence of the effectiveness of service learning on students' academic success, personal and social skills, and risk behaviour. However, the evidence was inconclusive. We found only few randomised controlled trials and the risk of bias in the included non‐randomised studies was very high. All available evidence used in the data synthesis was US‐based. The majority of studies available for meta‐analysis reported on a very limited number of outcomes; in particular few reported results on students' academic success even though the outcome was collected. Further, the majority of studies used in the meta‐analyses reported implementation problems. These considerations point to the need for more rigorously conducted studies performed outside the United States, reporting a larger number of outcomes. It would be natural to consider conducting a series of randomised controlled trial with specific allocation to implementation of high‐quality service learning as guided by the eight standards: (1) Meaningful service, (2) Link to curriculum, (3) Reflection, (4) Diversity, (5) Youth voice, (6) Community partnerships, (7) Progress monitoring and (8) Sufficient duration and intensity. Specific attention would also have to be paid to stringency in terms of conducting a well‐designed randomised trial with low risk of bias and ensuring that the sample sizes are large enough to enable sufficient power.

## 1 PLAIN LANGUAGE SUMMARY

### 1.1 Evidence of service learning in primary and secondary education is inconclusive

School‐based service learning is a teaching strategy that explicitly links community service to academic instruction. In this review, we aimed to find evidence of the effectiveness of service learning on students' academic success, personal and social skills, and risk behaviour. However, the evidence is inconclusive because of the small number of studies.

### 1.2 What is this review about?

Service learning is distinctive from traditional voluntarism or community service in that it intentionally connects service activities with curriculum concepts and includes structured time for reflection.

This review examines the evidence of impact of service learning on students' ‘neither employed, nor in education or training’ (NEET) status after compulsory schooling, academic success personal and social skills, and risk behaviour of students in primary and secondary education (from Kindergarten to Grade 12).

**What is the aim of this review?**

This Campbell systematic review examines the effects of service learning on academic success in students in primary and secondary education. The review summarises evidence from 10 studies undertaken in the USA that involved over 8,000 service learning participants in total.

### 1.3 What studies are included?

Included studies had to examine the impact of service learning in primary and secondary education. Studies had to have a comparison group.

Thirty‐seven studies analysing 30 different populations were identified. Of these, only 10 studies, analysing nine different populations, could be used in the data synthesis.

The studies were all from the USA. There were eight randomised controlled trials (RCTs) reported in nine studies and one non‐randomised study. The studies contained data for over 8,000 service learning participants.

### 1.4 What is the effect of service learning on academic success in students in primary and secondary education?

The evidence was inconclusive. The majority of studies available for meta‐analysis reported on a very limited number of outcomes; in particular, few reported results on students' academic success even though the outcome was collected. At most, the results from three studies could be pooled in a single meta‐analysis. Further, the majority of studies used in the meta‐analyses reported implementation problems.

There was no evidence of adverse effects.

### 1.5 What do the findings of the review mean?

The current landscape of research on service learning in primary and secondary education (grades kindergarten to 12) in general education shows that it has yet to be evaluated thoroughly. The evidence was inconclusive because too few studies reported results on the same type of outcome.

Furthermore, all the available evidence used in the data synthesis was US‐based, and so the findings may not be generalisable to other settings and systems outside the USA.

Also, the majority of studies used in the meta‐analyses reported implementation problems.

These considerations point to the need for more rigorously conducted studies reporting a larger number of outcomes.

### 1.6 How up‐to‐date is this review?

The review authors searched for studies published up to October 2020.

## 2 BACKGROUND

### 2.1 Description of the condition

Completion of upper secondary education marks the minimum threshold for successful labour market entry and continued employability as suggested by the Organisation for Economic Co‐operation and Developments (OECD's) annual indicators on education and associated labour market outcomes (OECD, 2015). On average across OECD countries, unemployment risk of younger adults (25–34 year‐olds) who have not completed upper secondary education is almost double the risk of those with higher educational qualifications (upper secondary and postsecondary non‐tertiary education). A maintained focus on completion rates is necessary. Even though enrolment rates among 15–16‐year‐olds (i.e., those typically in upper secondary programmes) are high; at least 95% on average across OECD countries in 2015 (OECD, 2018); far from all students graduate. According to OECD, only approximately 75% of students who had enroled had graduated after two years from the theoretical end date of the programme. Further, of the students who had not graduated, 80% were no longer enroled in education.

Many countries set specific targets for the completion rates of upper secondary education. For example, the countries in the European Union (EU) agreed on a 10‐year strategy proposed by the European Commission on 3 March 2010 for advancement of the economy of the EU (Europe 2020). One of the main targets is to reduce the share of early school‐leavers to 10% from the (at that time) current 15% and increase the share of the population aged 30–34 having completed tertiary from 31% to at least 40% (European Commission, 2010). Some countries go even further: Denmark has for example a specific target that upper secondary completion rates should be 95% and tertiary enrolment and completion rates should be 60% by 2020 (OECD, 2013a).

Not only graduation rates are important, the quality of the education received also matters for the educational prospects of young people and successful entry into the labour market. The shares of neither employed nor in education or training (NEET) are negatively related to the skill levels among young people (OECD, 2017a). The OECD's Programme for International Student Assessment (PISA) tests students near the end of their compulsory education (usually around age 15) on their reading ability, their skills in math and level in sciences. In general, the higher the percentage of low‐performing 15‐year‐old students in PISA, the higher the percentage of NEETs among 15–19 year‐olds (OECD, 2017a).

Having acquired some knowledge and skills that are essential for full participation in modern societies, particularly in reading, mathematics and science may be more reliable predictors of economic and social well‐being than the number of years spent in school or in post‐formal education (OECD, 2016). Research based on the 2012 Survey of Adult Skills (PIAAC) finds that poor proficiency in numeracy and literacy limits access to rewarding and well‐paid jobs, and in addition is linked to poorer health and less social and political participation (OECD, 2013b).

There is, for these reasons, a significant interest in information about effective interventions to increase academic achievement and enhance educational prospects. The review we conducted focused on service learning in primary and secondary education. Service Learning is curriculum‐based community service that integrates classroom instruction with community service activities. The connection with specific courses and having clearly stated learning objectives is what distinguishes service learning from other forms of volunteer work. Service learning should ‘address real community needs in a sustained manner over a period of time; and assist students in drawing lessons from the service through regularly scheduled, organised reflection of critical analysis activities, such as classroom discussions, presentations, or directed writing’ (Pritchard, 2002, p. 20). Well‐designed service‐learning activities can deepen learning and foster higher‐order thinking skills by providing students with opportunities to apply their learning to a challenging situation or problem in their community.

The development of service learning as a pedagogical method that integrates community service into the course curriculum began in the 1970s, primarily in the USA (Spring et al., 2008). In the nineties, service learning became institutionalised in public education in the United States (Peterson & Seligman, 2004). In 1990, the National and Community Service Act created Serve America (later named Learn and Serve America), which was a federal programme dedicated to providing grants and other supports for service learning activities in schools and community‐based organisations [1]. Further, in 1994, service learning became a recognised method for meeting the aims of federal school funding (included in the Elementary and Secondary Education Act). In addition to these federal policies, several states and school districts mandated the incorporation of service learning into the course curriculum (Education Commission of the States, 2014; Spring et al., 2008).

Service learning is not yet as widespread in the rest of the world. However, the OECD‐project ‘Innovative Learning Environments’ mentions service learning as a pedagogical method to put learners at the centre (the first of the seven principles of learning needed to redesign the learning environments to meet the challenges of the 21st century) (Dumont et al., 2010). According to Furco (2010) ‘service‐learning is one of the fastest growing educational initiatives in contemporary primary, secondary and postsecondary education’ (p. 228). Outside the USA, service‐learning initiatives are part of the education systems of Argentina, Columbia and Singapore (Chua, 2010; Ierullo, 2016; Perold & Tapia, 2008). Argentina hosts the Latin American Center for Service‐Learning (CLAYSS) which was created in 2002 to support students, educators, and community organisations in the development of service‐learning projects in Latin America. Service learning is not part of any educational policy in Europe, although the EU recognises service learning as a way of achieving citizenship education (European Commission/EACEA/Eurydice, 2017). Service learning is however emerging in many European countries including Germany, Ireland, Italy, Spain and the United Kingdom (Furco, 2010), and currently CLAYSS is assisting in the creation of the Central and Eastern European Service‐Learning Network (Regina & Ferrara, 2017).

In several European nations there are organisations (non‐profit community‐based) with programmes dedicated to providing supports for service learning activities in schools (Luna, 2012): Lernen durch Engagement in Germany, Center for Frivilligt Socialt Arbejde in Denmark, Lernen durch Engagement in Switzerland, Noi‐orizonturi in Romania, MOVISIE in the Netherlands and Fundación Tomillo in Spain.

### 2.2 Description of the intervention

School‐based service‐learning is a teaching strategy that explicitly links community service to academic instruction (Billig, 2000). In the United States, 'service‐learning' is an official term used by policymakers and educational leaders. Service‐learning is distinctive from traditional voluntarism or community service in that it intentionally connects service activities with curriculum concepts and includes structured time for reflection. Service‐learning is not an add‐on to an existing curriculum, a requirement of minimum hours of service to graduate or service assigned as punishment. Rather, students are required to use academic knowledge and skills to address genuine community needs. A clarifying example is given by the National Youth Leadership Council (https://www.nylc.org/page/our-philosophy):

Picking up trash on a river bank is service.

Studying water samples under a microscope is learning.

When science students collect and analyse water samples, document their results, and present findings to a local pollution control agency—that is service‐learning.

Service learning programmes can take many forms and are very diverse in content. However, a common set of elements are critical for a successful implementation of service learning. The National Youth Leadership Council and RMC Research Associates have developed a set of eight quality service‐learning standards (the K‐12 Service‐Learning Standards for Quality Practice) with input from youth, teachers, administrators, youth agencies, policymakers, community members, and other stakeholders. The standards are:

- Meaningful service: Service‐learning actively engages participants in meaningful and personally relevant service activities.
- Link to curriculum: Service‐learning is intentionally used as an instructional strategy to meet learning goals and/or content standards.
- Reflection: Service‐learning incorporates multiple challenging reflection activities that are ongoing and that prompt deep thinking and analysis of oneself and one's relationship to society.
- Diversity: Service‐learning promotes understanding of diversity and mutual respect among all participants.
- Youth voice: Service‐learning provides youth with a strong voice in planning, implementing and evaluating service‐learning experiences with guidance from adults.
- Partnerships: Service‐learning partnerships are collaborative, mutually beneficial, and address community needs.
- Progress monitoring: Service‐learning engages participants in an ongoing process to assess the quality of implementation and progress towards meeting specified goals, and uses results for improvement and sustainability.
- Duration and intensity: Service‐learning has sufficient duration and intensity to address community needs and meet specified outcomes.

The complete document can be accessed at https://www.nylc.org/page/standards.

### 2.3 How the intervention might work

Service learning, by connecting education to real world issues and allowing students to address problems they identify, may be particularly efficacious as it increases engagement and motivates students, in particular students who might not respond well to more traditional teaching methods (see, e.g., Bridgeland et al., 2008; Kraft & Wheeler, 2003; Scales & Roehlkepartain, 2005).

Motivation for learning and school engagement play a critical role in students' academic success (e.g., Fan & Wolters, 2014; Skaalvik & Valas, 1999). Motivated students tend to do better at school. According to OECD, students who are among the most motivated score the equivalent of more than one school year higher in PISA than the least‐motivated students and motivation is further positively related to life satisfaction (OECD, 2017b).

Theoretically, Kolb's (1984) model of experiential learning is often referred to as the foundation for understanding how service‐learning might work. Experiential learning theory defines learning as ‘The process whereby knowledge is created through the transformation of experience’ and knowledge is defined as: ‘a transformation process being continuously created and recreated, not an independent entity to be acquired or transmitted’ (Kolb, 1984, p. 38). Kolb further suggests that experiential approaches to learning such as service‐learning are better at accommodating learners with different learning styles than traditional didactic approaches such as classroom‐based teaching.

Experiential learning is inspired by pragmatist philosopher John Dewey's six‐step process of experiential logical inquiry. According to Dewey the six steps are: (1) encountering a problem, (2) formulating a problem or question to be resolved (3) gathering information which suggests solutions (4) making hypotheses (5) testing hypotheses, and (6) making warranted assertions (Dewey, 1938; Giles & Eyler, 1994; Kolb, 1984). Kolb's (1984) model comprises these steps into a four stage experiential learning cycle involving: *Concrete Experiences, Reflective Observation, Abstract Conceptualization* and *Active Experimentation* (Cone & Harris, 1996; Kolb, 1984). Based on this conception, students participating in service‐learning are engaged in a cycle in which their work in the community promotes written and/or oral reflection. Under the guidance of teachers or instructors, reflective work may be used to form abstract concepts and generate hypotheses, which may then be cycled back into further concrete experiences. According to Kolb this way of learning allows a variety of students with different learning styles and abilities to develop and integrate their skills (Cone & Harris, 1996).

Service‐learning provides an opportunity for students to move between perceiving new information through experiencing the concrete, tangible, felt qualities of the world within the community and taking hold of new information through abstract conceptualization, thinking and analysing. The pattern in which a learner moves between these levels of experience are thought to reflect an individual learning style, and service‐learning is thought to allow each student to move between the levels in a way consistent with their own learning style (Kolb et al., 2002).

Another strand of theory which offers a potential understanding of the theory of change behind service‐learning is *Situated Learning*. The term ‘situated learning’ refers to learning that occurs within a particular and authentic context through the individual's social participation. Rather than focusing on learning as a primarily cognitive process involving a number of tasks, situated learning theorists study the process in which individuals become new members of a learning community. According to the theory newcomers within a learning community move from a state of legitimate peripheral participation to full participation through a process that involves continuous negotiation, collaboration, and reflection (Wolfson & Willinsky, 1998).

In their often cited work: ‘Situated Learning: Legitimate Peripheral Participation', Lave and Wenger (1991) focus on acquisition of skills and knowledge that takes place outside traditional schooling within communities of practice. Based on an ethnographic investigation of traditional and nontraditional apprenticeships in Mexico, Liberia and the United States, Lave and Wenger propose that learning should not be viewed as the mere transmission of knowledge but as a distinctly embedded and active process. Learning is thus perceived as a contextualised process in which content is learned through doing activities. Furthermore, Lave and Wenger suggest that motivation too is ‘situated', as learners are naturally motivated by their growing value of participation (Lave & Wenger, 1991). Based on this approach students participating in service‐learning inherently become motivated to learn as this enables them to move from being novices to becoming full participants within the learning community. Furthermore, students participating in service‐learning may become motivated as they experience how their own participation increases in value as they progress from being newcomers towards the center of the community of practice.

In situated learning the construction of meaning is seen as being tied to specific contexts and purposes. For students participating in service‐learning this may be particularly important, as service learning may enable them to socially construct meaning which makes learning matter beyond school.

#### 2.3.1 Service‐learning as way to promote positive youth development and leadership

In a review of youth development outcomes in out of school settings, Eccles and Gootman (2002) concluded, that there are four areas of assets that facilitate positive youth development: *physical, intellectual, psychological/emotional*, and *social*. Although strong assets in one domain can compensate for weak assets in another, optimal youth development is facilitated when a young person requires assets in all areas (Eccles & Gootman, 2002). This view is highly consistent with the theory of change in service‐learning, as the goal in service‐learning is not restricted to teaching students a specific predefined curriculum. Through their concrete work, students may expand both their physical skills and intellectual knowledge, and they may improve their social and emotional well‐being by participating in a community.

Another way of conceptualising the theory of change in service‐learning is found in van Linden and Fertman's (1998) description of the three stages of youth leadership development. According to Fertman and Van Linden (1999) all students have leadership potential. Leaders are defined as individuals ‘who think for themselves, communicate their thoughts and feelings to others, and help others understand and act on their own beliefs. They influence others in an ethical and socially responsible way’ (Fertman and Van Linden, 1999, p. 10). There are three stages of youth leadership development: awareness, interaction, and mastery (van Linden & Fertman, 1998). They are sequential but fluid. Adolescents may move from one stage to the next, only to return to the previous stage when they encounter a new situation, and this process may be facilitated by participating in service‐learning, in which the students are confronted with real world problems which may increase their awareness of social and ethical dilemmas.

Finally, the theory of change behind service learning shares similarities with the concept authentic learning (Slavkin, 2004). Authentic learning refers to a pedagogical practice or strategy in which teachers share responsibility with parents and students. Authentic learning seeks to motivate students who are demotivated by traditional classroom activities by creating activities and assignments that encourage students to reflect upon their classroom and community as well as encourage them to improve their citizenry. In authentic learning, the assumption is that teachers should move beyond only installing one core of knowledge, and instead help students through guiding the practice of learning (Slavkin, 2004). Thus, service‐learning may be seen as a transformative educational practice, empowering students to do their best work through realisingrealizing the importance of being active citizens (Slavkin, 2007).

### 2.4 Why it is important to do this review

Two systematic reviews with meta‐analyses are found in Conway et al. (2009) and Celio et al. (2011), both performing searches up to spring 2008. The review by Conway et al. (2009) analysed four outcomes: academic, personal, social, and citizenship outcomes. Many of the included studies did not have control groups. They furthermore included studies of community service or volunteerism as well as service learning without distinguishing between these very different types of interventions (except in a moderator analysis), participants were not limited to primary and secondary education (although all results were shown separately for grade kindergarten to 12 students but without distinguishing between community service or volunteerism and service learning).

The review by Celio et al. (2011) required included studies to analyse service learning using a control group, but participants were not limited to primary and secondary education. Five outcome areas were analysed: attitudes towards self, attitudes towards school and learning, civic engagement, social skills, and academic achievement. Separate results for primary and secondary education (grades kindergarten to 12) was only shown for the overall effect, that is, the mean of the five outcome domains attitudes towards self, attitudes towards school and learning, civic engagement, social skills, and academic achievement.

Besides being up to date, the major differences between these two systematic reviews and the current review are that we focused on service learning for primary and secondary education, only included studies with a control group, all relevant outcomes areas were analysed separately, and we took into consideration the dependencies between effect sizes.

In addition, there are several literature reviews of studies conducted in the United States (Billig, 2000, 2002, 2003, 2004). None of them is a systematic review and no data synthesis is performed in any of them. The review we performed differed in substantial ways from these existing reviews. It is systematic and several meta‐analyses were conducted.

## 3 OBJECTIVES

The main objective of this review is to answer the following research question: What are the effects of service learning on academic success, NEET status, personal and social skills, and risk behaviour of students in primary and secondary education (grades kindergarten to 12)?

Further, we wanted to investigate the following factors with the aim of explaining potential observed heterogeneity: study‐level summaries of participant characteristics (e.g., studies considering a specific gender, age or socioeconomic level or studies where separate effects for girls/boys, primary school/secondary school or low/high socioeconomic status are available) and quality of the service learning programme according to the standards as outlined in section *The intervention*.

## 4 METHODS

### 4.1 Criteria for considering studies for this review

#### 4.1.1 Types of studies

The project followed standard procedures for conducting systematic reviews using meta‐analysis techniques. The systematic review protocol (Filges et al., 2021) was published in June 2021. The protocol is available at: https://doi.org/10.1002/cl2.1157.

To summarise what is known about the possible causal effects of service learning, we included all study designs that use a control group, that is, a group of students not participating in service learning. The control group could be offered treatment as usual or an alternative treatment.

The study designs eligible for the review were:

1. Randomised and quasi‐randomised controlled trials: allocated at either the individual level or cluster level (e.g., class/school/geographical area etc.).
2. Non‐randomised studies: service learning has occurred in the course of usual decisions, the allocation to service learning and no service learning is not controlled by the researcher, and there is a comparison of two or more groups of participants (i.e., at least a treated group and a control group).

Studies using single group pre‐post comparisons were not eligible. Non‐randomised studies using an instrumental variable approach were not eligible—see the Supporting Information Appendix [*Justification of exclusion of studies using an instrumental variable (IV) approach*] for our rationale for excluding studies of these designs. A further requirement of all types of studies (randomised as well as non‐randomised) was that they were able to identify an intervention effect, i.e., they should have assigned at least two units (e.g., students, teachers, classes, or schools) to the treatment group and at least two units to the control group. Studies where, for example, the treatment was given to teachers in one school only and the comparison group was teachers at another school (or more schools for that matter) cannot separate the treatment effect from the school effect and can thus not identify an intervention effect. Even within schools, organisation of teachers in teacher teams may mean that randomisation would have to be at the teacher team level to be able to avoid a situation of not being able to separate teacher‐level treatment effect from teacher‐team effect. Further, studies must also satisfy specific risk of bias criteria before contributing to the data synthesis (see Assessment of risk of bias in included studies).

#### 4.1.2 Types of participants

Children in primary and secondary education (grades kindergarten to 12) in general education were eligible.

The included grades correspond to primary and secondary school, defined as the first two steps in a three‐tier educational system consisting of primary education, secondary education, and tertiary or higher education. The number of years a child attend primary schooling varies across the OECD countries, though most often primary schooling is K‐6 or K‐9 after which secondary education begins (e.g., in the form of high school). The former is the case for instance in France, Spain, Japan, UK, and most parts of Australia, and the second is the case for school systems in countries such as Italy, Turkey, Sweden and Denmark. The eligible age range differed between countries, and sometimes between states within countries. Typically, ages range from 5 to 7 to 11–13 in primary school and from 12 to 14 to 17–19 in secondary school. In some countries, kindergarten can however refer to preschool programmes outside primary school and include ages down to 2 years. Service learning targeting such populations were excluded; that is, kindergarten had to be considered a part of primary school for a study to be included.

Studies that met inclusion criteria were accepted from all countries. We excluded children in home school and in preschool programmes.

#### 4.1.3 Types of interventions

Service Learning is a curriculum‐based community service that integrates classroom instruction (such as classroom discussions, presentations, or directed writing) with community service activities. Service learning may be mandatory or voluntary, and should have service activities that take place outside the classroom. It should take place in the community including the school as part of the community. Service learning is organised in relation to an academic course or curriculum and has clearly stated learning objectives. Service learning should address real community needs and involve students in drawing lessons from the service through regularly scheduled, organised reflection or critical analysis. Community service or extracurricular activities that do not integrate classroom instruction were excluded.

#### 4.1.4 Types of outcome measures

##### Primary outcomes

The primary focus was on measures of academic success and NEET status (neither employed nor in education or training post compulsory school). The eligible primary outcomes were:

- scores on students' achievement tests
- attendance
- drop‐out
- employment, education, training (NEET status)

Concerning scores on students' achievement tests, only standardised measures were eligible, such as, norm‐referenced tests (e.g., Gates‐MacGinitie Reading Tests and Star Math), state‐wide tests (e.g., Iowa Test of Basic Skills), national tests (e.g., National Assessment of Educational Progress) and measures of global academic performance (e.g., Woodcock‐Johnson III Tests of Achievement, Stanford Achievement Test, Grade Point Average).

Although we did not expect to find (and did not find any) studies reporting follow‐up outcomes in the long run (post compulsory school), NEET status was planned as a primary outcome.

Concerning students' achievement tests, standardised measures reported in the included studies were statewide tests obtained from school records and the Metropolitan Achievement Test. Attendance, obtained from school records, was reported in two studies. No studies reported drop out or NEET status.

##### Secondary outcomes

A secondary focus was on measures of personal and social skills (including self‐perception/self‐confidence and attitudes towards helping others), and risk behaviour (such as drug and alcohol use, violent behaviour, sexual risk taking; measured by self‐reports or reports by authorities, administrative files, registers).

Concerning personal and social skills, only valid and reliable outcomes that had been standardised on a different population (and is ‘objective', i.e., not ‘experimenter‐designed') were included. Examples of valid outcomes are measures from the Social Skills Rating System (SSRS; Gresham & Elliott, 1990) or the revision of the SSRS, called the Social Skills Improvement System‐Rating Scales (SSIS‐RS; Gresham & Elliott, 2008) and the Academic Competence Evaluation Scales (ACES) (DiPerna & Elliott, 1999).

Only two standardised personal and social skills outcomes were reported in more than one of the included studies, namely self‐esteem and locus of control. Self‐esteem was measured by The Self‐Esteem Questionnaire (Dubois 1996) and The Scholastic subscale from the Secondary‐Level of the Self‐Appraisal Inventory and the Self Observation Scales (Junior High Level, Form C). Locus of control was measured by the Connell scale, the Children's perception of control (a subscale of the Research/Assessment Package for Schools), The Scholastic subscale from the Secondary‐Level of the Self Appraisal Inventory, the Self Observation Scales (Junior High Level, Form C) and The Nowicki‐Strickland Locus of Control Scale for Children (Nowicki & Strickland, 1973). In addition, a number of other personal and social skills outcomes (measured by a variety of different standardised scales) were reported but could not be meta analysed as each outcome was reported in only one study (see Table 8).

Concerning risk behaviour seven studies (reporting on five trials) reported on different measures of sexual risk taking (engagement in unprotected sex and ever been/caused someone to be pregnant) measured by self‐reports. In addition, a number of other risk behaviours were reported but could not be meta analysed as each outcome was reported in only one study (see Table 8).

Studies were only included if they considered at least one of the primary or secondary outcomes. If it was not clear from the description of outcome measures in the studies whether they are standardised, we used electronic sources to determine whether a measure was standardised or not. We did not consider measures where researchers had picked a subset of questions from a standardised measure.

No potential adverse effects have been evaluated in any included studies.

###### 4.1.4.1 Duration of follow‐up

Time points planned for measures were:

- 0–1‐year follow up
- 1–2‐year follow up
- More than 2‐year follow up

All measures were taken at postintervention (although one study only reported results at a 1‐year follow up and not the result of measures taken postintervention). In addition, four studies reported a follow‐up of approximately 1 year.

###### 4.1.4.2 Types of settings

We included classes in primary and secondary education (grades kindergarten to 12) in regular private, public or boarding schools. Home‐schools were excluded.

### 4.2 Search methods for identification of studies

We implemented a wide range of search methods and strategies to maximise coverage of relevant references, while simultaneously attempting to reduce different types of bias related to publication and dissemination systems. The different strategies and methods are presented below.

#### 4.2.1 Electronic searches

##### 4.2.1.1 Selection of bibliographical databases

We selected bibliographical databases that cover journals from different academic disciplines relating to the topic of the review. We also selected databases with a general academic scope, to ensure coverage beyond the expected academic fields. We selected the follow databases (the platform used for the search is in parenthesis):

- ERIC (EBSCO)
- CINAHL (EBSCO)
- Academic Search Premier (EBSCO)
- EconLit (EBSCO)
- PsycINFO (EBSCO)
- SocIndex (EBSCO)
- Teacher Reference Center (EBSCO)
- Sociological Abstracts (ProQuest)
- Science Citation Index Expanded (Web Of Science)
- Social Sciences Citation Index (Web Of Science)

##### 4.2.1.2 Example of a search string

Below is an example of a search‐string utilised to search SocIndex through the EBSCO‐platform. This search string was modified in accordance with the search interface, syntax and subject terms for each of the above standing databases.

**Table:** 

Search	Search Terms	Results
S8	S4 AND S7	1209
S7	S5 OR S6	340,514
S6	AB student* OR AB pupil* OR AB school* OR AB adolescen*	325,776
S5	TI student* OR TI pupil* OR TI school* OR TI adolescen*	125,153
S4	S1 OR S2 OR S3	1777
S3	AB ‘service learning’ OR AB ‘experiential learning’ OR AB ‘school community program*'	1371
S2	TI ‘service learning’ OR TI ‘experiential learning’ OR TI ‘school community program*'	682
S1	DE ‘SERVICE learning’	671

##### 4.2.1.3 Description and rationale for search terms and facets, and sensitivity of the search string

The search string was designed to balance sensitivity and precision. The search string contains two aspects related to the inclusion criteria of the review. To keep the search string sufficiently sensitive, we searched each aspect in either title, abstract, or subject terms.

- Search 1–3 covers the intervention
- Search 5–7 covers the population
- Search 8 combines the two aspects

A full report on the search strings and results for each database search can be found in the Supporting Information Appendix 5.

After finishing the review, it was brought to our attention by an anonymous referee that in some school districts in the United States, service‐learning practice is often cast as 'project‐based learning'. in which the projects students conduct are situated in the community and are designed to meet a community need; implying some of them may meet the inclusion criteria's of our review. As 'project‐based learning' was not part of the search terms used in the bibliographic databases or in the grey literature search, some studies of service learning may not have been identified in our search. However, we believe the risk of not identifying relevant studies is very small. We have looked through a review on project‐based learning brought to our attention by a referee (Kingston, 2018). We screened the 20 studies included in the review and none of them analysed a service learning intervention and did not meet the inclusion criteria's for our review. Furthermore, we have located a study on project‐based service learning (PBSL) saying that ‘SL is generally conducted via PBL; thus, this approach is often colloquially referred to as *project‐based service learning* (PBSL) by its practitioners’ (p. 535). The same argument can be found in Furco, 2003. Thus, we believe that any studies on PBSL would have been identified by the search terms applied in our search strategy.

*Limitations of the search string*

No limitations were implemented during the database searches.

#### 4.2.2 Searching other resources

We searched a range of web‐based resources to identify references that were either unpublished, not in English, or both.

Some resources listed contains multiple types of unpublished literature, as well as published references. The resources we searched are listed under the category of literature that is most prevalent in the resource.

*Searches for working papers and conference proceedings in English*

- SSRN Working Papers—http://www.ssrn.com
- Open Grey—http://opengrey.eu

*Searches for dissertation and theses in English*

- ProQuest Dissertations & Theses Global (ProQuest)
- EBSCO Open Dissertations (EBSCO)

*Searches for reports in English*

- Education Commission of the States—https://www.ecs.org
- National Youth Leadership Council—https://www.nylc.org
- Search Institute—https://www.search-institute.org
- Manpower Demonstration Research Corporation—https://www.mdrc.org
- American Institutes for Research—https://www.air.org
- RAND—https://www.rand.org
- Mathematica—https://www.mathematica.org
- CIRCLE—https://civicyouth.org/ResearchTopics/research-topics/service-learning

*Searches for ongoing studies in English*

- Google Scholar—https://scholar.google.com
- Google searches—https://www.google.com

*Searches for working papers, conference proceedings, dissertations and theses on other languages*

- Danish National Research Database—http://www.forskningsdatabasen.dk/en

*Searches for reports on other languages*

- Google Scholar—https://scholar.google.com
- Google searches—https://www.google.com

##### Hand searches

We implemented hand searches in key journals to identify references that were poorly indexed in the bibliographical databases, as well as covering references that was published in a journal, but not yet indexed in the bibliographical databases during the search process.

Our selection of journals to hand search was based on the frequency of the journals in our pilot‐searches for designing the search‐strings in the protocol phase. Journals with the highest frequence of references in the pilot searches were selected for hand search and a few journals were added due to peer referee suggestions. We searched the following journals:

- *Journal of Experiential Education* (2019–2021)
- *Journal of Adolescence* (2019–2021)
- Journal of Early Adolescence (2019‐2021)
- *Journal of Prevention and Intervention in the Community* (2019–2021)
- *The International Journal of Research on Service‐Learning and Community Engagement* (2013–2017)

We further searched the contents of the books published in the *Advances in Service‐Learning Research Series*.

We had further planned to search the journal *International Journal of Research on Service‐Learning in Teacher Education* which we could not access because the website was undergoing technical maintenance.

##### Citation‐tracking and snowballing

Systematic reviews and key references identified during the search process was citation tracked to identify additional relevant references. The systematic reviews and key references selected for citation tracking is listed in the appendix.

### 4.3 Data collection and analysis

#### 4.3.1 Selection of studies

**Figure 1:**
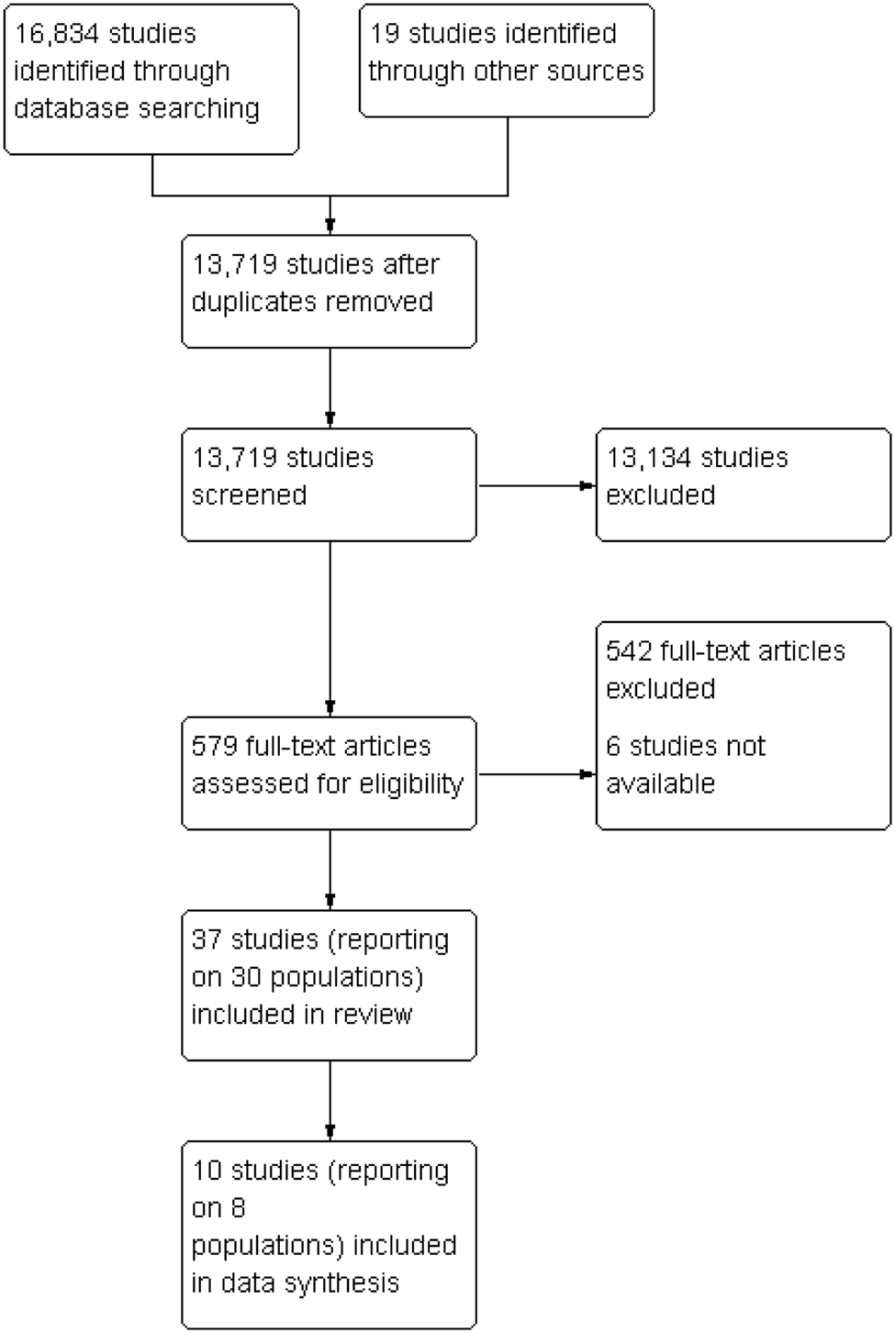
Flow diagram

Under the supervision of the review authors, two review team assistants first independently screened titles and abstracts to exclude studies that were clearly irrelevant. Studies considered eligible by at least one assistant or studies with insufficient information in the title and abstract to judge eligibility, were retrieved in full text. The full texts were then screened independently by two review team assistants under the supervision of the review authors. Any disagreement of eligibility was resolved by the review authors. Exclusion reasons for studies that otherwise might be expected to be eligible were documented and presented in section *Characteristics of excluded studies*. The study inclusion criteria were piloted by the review authors (see Supporting Information Appendix 1). The overall search and screening process is illustrated in Figure 1. None of the review authors were blind to the authors, institutions, or the journals responsible for the publication of the articles.

#### 4.3.2 Data extraction and management

Independent screening and deduplication of identified records was carried out in EPPI‐Reviewer 4 version 4.12.0.0.

Two review authors independently coded and extracted data from included studies.

A coding sheet was piloted on several studies and revised as necessary (see Supporting Information Appendix). Disagreements were minor and were resolved by discussion. Data and information was extracted on: available characteristics of participants, intervention characteristics and control conditions, research design, sample size, risk of bias and potential confounding factors, outcomes, and results. Extracted data was stored electronically. The analysis was conducted in RevMan5.

Extracted numerical and descriptive data, and the risk of bias assessments described in the next section, can be found at https://osf.io/v8ceq/.

#### 4.3.3 Assessment of risk of bias in included studies

We assessed the risk of bias in randomised studies using Cochranes revised risk of bias tool, RoB 2 (Higgins et al., 2019).

The tool is structured into five domains, each with a set of signalling questions to be answered for a specific outcome. The five domains cover all types of bias that can affect results of randomised trials.

The five domains for individually randomised trials are:

(1) bias arising from the randomisation process;
(2) bias due to deviations from intended interventions (separate signalling questions for effect of assignment and adhering to intervention);
(3) bias due to missing outcome data;
(4) bias in measurement of the outcome;
(5) bias in selection of the reported result.

For cluster‐randomised trials, an additional domain was included ((1b) Bias arising from identification or recruitment of individual participants within clusters). We used the latest template for completion (the version of 15 March 2019 for individually randomised parallel‐group trials and 20 October 2016 for cluster randomised parallel‐group trials). In the cluster randomised template, however, only the risk of bias due to deviation from the intended intervention (effect of assignment to intervention; intention to treat ITT) was present and the signalling question concerning the appropriateness of the analysis used to estimate the effect was missing. Therefore, for cluster randomised trials we only used the signalling questions concerning the bias arising from identification or recruitment of individual participants within clusters from the template for cluster randomised parallel‐group trials; otherwise we used the template and signalling questions for individually randomised parallel‐group trials.

We assessed the risk of bias in non‐randomised studies, using the model ROBINS–I, developed by members of the Cochrane Bias Methods Group and the Cochrane Non‐Randomised Studies Methods Group (Sterne et al., 2016a). We used the latest template for completion (the version of 19 September 2016).

The ROBINS‐I tool is based on the Cochrane RoB tool for randomised trials, which was launched in 2008 and modified in 2011 (Higgins et al., 2011). The tool covers seven domains (each with a set of signalling questions to be answered for a specific outcome) through which bias might be introduced into non‐randomised studies:

(1) bias due to confounding
(2) bias in selection of participants
(3) bias in classification of interventions
(4) bias due to deviations from intended interventions;
(5) bias due to missing outcome data;
(6) bias in measurement of the outcome;
(7) bias in selection of the reported result.

The first two domains address issues before the start of the interventions and the third domain addresses classification of the interventions themselves. The last four domains address issues after the start of interventions and there is substantial overlap for these four domains between bias in randomised studies and bias in non‐randomised studies trials (although signalling questions are somewhat different in several places, see Sterne et al., 2016b and Higgins et al., 2019).

Randomised study outcomes are rated on a ‘Low/Some concerns/High’ scale on each domain; whereas non‐randomised study outcomes are rated on a ‘Low/Moderate/Serious/Critical/No Information’ scale on each domain. The level ‘Critical’ means: the study (outcome) is too problematic in this domain to provide any useful evidence on the effects of intervention, and it is excluded from the data synthesis. The same critical level of risk of bias (excluding the result from the data synthesis) is not directly present in the RoB 2 tool, according to the guidance to the tool (Higgins et al., 2019).

In the case of a RCT, where there is evidence that the randomisation has gone wrong or is no longer valid, we planned to assess the risk of bias of the outcome measures using ROBINS‐I instead of RoB 2. Examples of reasons for assessing RCTs using the ROBINS‐I tool may include studies showing large and systematic differences between treatment conditions while not explaining the randomisation procedure adequately suggesting that there was a problem with the randomisation process; studies with large scale differential attrition between conditions in the sample used to estimate the effects; or studies selectively reporting results for some part of the sample or for only some measured outcomes. In such cases, differences between the treatment and control conditions are likely systematically related to other factors than the intervention and the random assignment is, on its own, unlikely to produce unbiased estimates of the intervention effects. Therefore, as ROBINS‐I allow for an assessment of for example confounding, we believe it is more appropriate to assess effect sizes from studies with a compromised randomisation using ROBINS‐I than RoB 2. If so, we would report this decision as part of the risk of bias assessment of the outcome measure in question. As other effect sizes assessed with ROBINS‐I, these effect sizes could have received a ′Critical′ rating and thus be excluded from the data synthesis.

We stopped the assessment of a non‐randomised study outcome as soon as one domain in the ROBINS‐I was judged as ‘Critical'.

‘Serious’ risk of bias in multiple domains in the ROBINS‐I assessment tool may lead to a decision of an overall judgement of ‘Critical’ risk of bias for that outcome, and it will be excluded from the data synthesis.

##### Confounding

An important part of the risk of bias assessment of non‐randomised studies is consideration of how the studies deal with confounding factors. Systematic baseline differences between groups can compromise comparability between groups. Baseline differences can be observable (e.g., age and gender) and unobservable (to the researcher; e.g., motivation and ‘ability'). There is no single non‐randomised study design that always solves the selection problem. Different designs represent different approaches to dealing with selection problems under different assumptions, and consequently require different types of data. There can be particularly great variations in how different designs deal with selection on unobservables. The ‘adequate’ method depends on the model generating participation, that is, assumptions about the nature of the process by which participants are selected into a programme.

A major difficulty in estimating causal effects of service learning on student outcomes is the potential endogeneity of service learning stemming from the decision process of introducing service learning as a pedagogical method. Not only do families choose neighbourhoods and schools, but principals and other administrators assign students to classrooms and teachers. Because these decision makers utilise information on students, teachers and schools, information that is often not available to researchers, the estimators are quite susceptible to biases from a number of sources.

As there is no universal correct way to construct counterfactuals for non‐randomised designs, we looked for evidence that identification was achieved, and that the authors of the primary studies justify their choice of method in a convincing manner by discussing the assumption(s) leading to identification (the assumption(s) that make it possible to identify the counterfactual). Preferably the authors should make an effort to justify their choice of method and convince the reader that the only difference between a treated student and a nontreated student is the treatment. The judgement is reflected in the assessment of the confounder unobservables in the list of confounders considered important at the outset (see Supporting Information Appendix *User guide for unobservables*).

In addition to unobservables, we had identified the following observable confounding factors to be most relevant: age and grade level, performance at baseline, gender and socioeconomic background. In each study, we assessed whether these factors had been considered, and in addition we assessed other factors likely to be a source of confounding within the individual included studies.

##### Importance of pre‐specified confounding factors

The motivation for focusing on age and grade level, performance at baseline, gender and socioeconomic background is given below.

Generally, development of cognitive functions relating to school performance and learning are age dependent, and furthermore systematic differences in performance level often refer to systematic differences in preconditions for further development and learning of both cognitive and social character (Piaget, 2001; Vygotsky, 1978).

Therefore, to be sure that an effect estimate is a result from a comparison of groups with no systematic baseline differences it is important to control for the students' grade level (or age) and their performance at baseline (e.g., reading level, math level).

With respect to gender it is well‐known that there exist gender differences in school performance (Holmlund & Sund, 2005). Girls outperform boys with respect to reading and boys outperform boys with respect to mathematics (Stoet & Geary, 2013). Although part of the literature finds that these gender differences have vanished over time (Hyde & Linn, 1988; Hyde et al., 1990), we find it important to include this potential confounder.

Students from more advantaged socioeconomic backgrounds on average begin school better prepared to learn and receive greater support from their parents during their schooling years (Ehrenberg et al., 2001). Further, Willms and Somers (2001) found that schools enroling students from higher socioeconomic backgrounds tended to have better infrastructures, more instructional materials, and better libraries. Finally, as outlined in the background section, students with socio‐economically disadvantaged backgrounds perform poorly in school tests (OECD, 2010). Therefore, the accuracy of the estimated effects of service learning will depend crucially on how well socioeconomic background is controlled for. Socioeconomic background factors are, for example, parents' educational level, family income, minority background, and so forth.

##### Effect of primary interest and important co‐interventions

We were mainly interested in the effect of starting and adhering to the intended intervention, that is, the treatment on the treated (TOT) effect. The risk of bias assessments was therefore in relation to this specific effect. The risk of bias assessments of both randomised trials and non‐randomised studies considered adherence and differences in additional interventions ('co‐interventions') between intervention groups.

Important co‐interventions we considered were interventions performed in school, during the regular school year, which are complementary to regular classes and school activities. They may be delivered individually (e.g., the Reading Apprenticeship programme or individual computer‐based training such as CogMed), in class (e.g., paired reading interventions or the Xtreme Reading programme), or in group sessions (e.g., the READ 180 programme).

##### Assessment

At least two review authors independently assessed the risk of bias for each relevant outcome from the included studies. We discussed all initial disagreements and were able to reach a consensus in all cases. We report the risk of bias assessment in risk of bias tables for each included study outcome in a supplementary document (available here: https://osf.io/v8ceq/).

#### 4.3.4 Measures of treatment effect

##### Continuous outcomes

All academic success, and personal and social skills outcomes were continuous measures. We calculated effects size with 95% confidence intervals where means and standard deviations were available, or alternatively from mean differences and standard deviations of the mean (whichever were available), using the methods suggested by Lipsey and Wilson (2001). If not enough information was available, we requested this information from the principal investigators. Hedges' *g* was used for estimating standardised mean differences (SMD).

##### Dichotomous outcomes

For dichotomous outcomes, we used odds ratios with 95% confidence intervals were available. One study reported dichotomous risk behaviour outcomes as probability differences. These effect sizes could not be pooled but is reported in the Supporting Information Appendix Table 8.

#### 4.3.5 Unit of analysis issues

##### Criteria for determination of independent findings

To account for possible statistical dependencies, we examined a number of issues: we assessed whether suitable cluster analysis was used, if assignment of units to treatment was clustered, whether individuals had undergone multiple interventions, whether there were multiple treatment groups, and whether several studies were based on the same data source.

###### 4.3.5.1 Clustered assignment of treatment

Errors in statistical analysis can occur when the unit of allocation differs from the unit of analysis. In cluster randomised trials, participants are randomised to treatment and control groups in clusters, either when data from multiple participants in a setting are included (creating a cluster within the school or community setting), or when participants are randomised by treatment locality or school. Non‐randomised studies may also include clustered assignment of treatment. Effect sizes and standard errors from such studies may be biased if the unit‐of‐analysis is the individual and an appropriate cluster adjustment is not used (Higgins & Green, 2011).

Two studies used in the meta‐analyses needed correction for treatment given in clusters. One study was a cluster randomised trial (Giacalone, 2004) and the other was an individually randomised group treatment trial (Santmire, 1999).

A study design where participants are individually randomised to treatment, but that treatment is delivered in a group setting, are known as *individually randomised group treatment* (IRGT) trials (Pals et al., 2008). The analysis in such a study design must correct for the fact that dependencies may arise between individuals that happen to receive the intervention in the same group. The analogy is the cluster randomised trial (CRCT) where clusters of participants are randomised to treatment. The analysis of CRCTs must correct standard errors for the dependencies among individual participants in clusters. The correction of IRGTs involves knowledge of the intra‐cluster correlation coefficient (ICC) and the (mean) group size in line with the correction of standard errors from CRCTs. With this in hand, the estimated standard errors can be corrected with a *design effect* (Hedges & Citkowicz, 2015).

However, none of the studies contained any information about estimates of the ICC or the within‐cluster and between‐cluster variances (the ICC is the ratio between the between‐cluster and the total variance). Neither did they inform about realised cluster sizes in the treatment and control groups. We therefore adjusted these two studies assuming equal cluster size (dividing the reported number of students with reported number of classes/teachers) in each condition, and we used an ICC of 0.10, which is very close to the mean of both reading and mathematics taken over Grades K‐6 in the pre‐test covariate models of tables 6 and 7 in Hedges and Hedberg (2007, pp. 72–73). We used the cluster corrected effect sizes in a sensitivity analysis.

###### 4.3.5.2 Multiple interventions groups and multiple interventions per individuals

There were no studies with multiple intervention groups or multiple interventions per individual.

###### 4.3.5.3 Multiple studies using the same sample of data

Three studies analysed the same data set (Melchior, 1995, 1998, 1999) and four studies analysed the same cluster randomised trial in Florida: Walsh‐Buhi (2016), Daley and Buhi (2015), Daley (2019), and Debate (2018).

We reviewed all studies, but in the meta‐analyses, we only included one estimate of the effect on a particular outcome from each sample of data to avoid dependencies between the ‘observations’ (i.e., the estimates of the effect) in the meta‐analyses. The choice of which estimate to include was based on our risk of bias assessment of the studies. We chose the estimate from the study that we judged to have the least risk of bias (primarily, Confounding bias). If two (or more) studies were judged to have the same risk of bias and one of the studies (or more) used a subset of a sample used in another study (or studies) we included the study using the full set of participants.

###### 4.3.5.4 Multiple time points

When the results were measured at multiple time points, each outcome at each time point were analysed in a separate meta‐analysis with other comparable studies taking measurements at a similar time point. All measures were taken at postintervention (although one study only reported results at the 1‐year follow‐up time and not the result of measures taken postintervention). In addition, four studies reported on an approximately 1 year follow‐up.

#### 4.3.6 Dealing with missing data

If a study did not include enough information to calculate an effect size and standard error, the review authors requested this information from the principal investigators. We contacted Alan Melchior, Principal Investigator for the study, who kindly provided the necessary information.

#### 4.3.7 Assessment of heterogeneity

Heterogeneity among primary outcome studies was assessed with *χ* ^2^ (*Q*) test, and the *I* ^2^, and *τ* ^2^ statistics (Higgins et al., 2003). Any interpretation of the *χ* ^2^ test was made cautiously on account of its low statistical power.

#### 4.3.8 Assessment of reporting biases

Reporting bias refers to both publication bias and selective reporting of outcome data and results. Here, we state how we planned to assess publication bias.

We planned to use funnel plots for information about possible publication bias however we did not find sufficient studies (Higgins & Green, 2011).

We were therefore unable to comment on the possibility of publication bias.

#### 4.3.9 Data synthesis

Meta‐analysis of outcomes were conducted on each metric (as outlined in section ‘Types of outcomes measures') separately.

When the effect sizes used in the data synthesis were odds ratios, they were log transformed before being analysed.

Studies that were coded Critical risk of bias were not included in the data synthesis.

All analyses were inverse variance weighted using random effects statistical models that incorporate both the sampling variance and between study variance components into the study level weights. All meta‐analyses were performed using Revman 5.4. The estimation of τ^2^ was the DerSimonian and Laird (1986) estimate (DerSimonian & Laird, 1986). Random effects weighted mean effect sizes were calculated using 95% confidence intervals.

One study, Moskowitz (1981), provided results separately by numerous subgroups [type of service (2), grade (2) and gender (2)].

To take into account the dependence between multiple effect sizes from the same study, we planned to apply a robust variance estimation (RVE) approach (Hedges et al., 2010). However, as there was not a sufficient number of studies to use RVE, in accordance with the protocol, we conducted the data synthesis using a synthetic effect size (the average) to avoid dependence between effect sizes with one exception. Although random effects models applied when synthetic effect sizes are involved perform better in terms of standard errors than do fixed effects models (Hedges, 2007a), the method overestimates the standard error. As means and standard deviations for numerous subgroups within each condition (eight) were reported in Moskowitz (1981) the number used for calculating the standard errors for each subgroup effect size was very low and the standard errors were most likely heavily overestimated. Further, as the subgroups represented a breakout on several sample characteristics (grade and gender) the full within‐group standard deviation is seriously underestimated because variability associated with the subgroup variable has been removed (variability in the outcome associated with grade and gender). We therefore used the formulas provided in Wilson (2015), section *3.19 Means and Standard Deviations with Subgroups* to calculate the standardised mean difference for the overall population. We calculated the weighted mean for each condition (i.e., treatment and control) as

$$\underline{X}=(\sum {\underline{X}}_{j}n_{j})/\sum n_{j},$$

where *j* represents each subgroup in each condition. The pooled within‐group standard deviation for each condition, ignoring any variance removed due to the subgroup variable, is calculated as

$$\surd \left(\sum s_{j}^{2}\left(n_{j}-1\right)/\sum \left(n_{j}-1\right)\right).$$

The subgroup variable is accordingly added back into the within group variance using the following formula:

$$\surd (\left(\sum s_{j}^{2}\left(n_{j}-1\right)/\sum \left(n_{j}-1\right)\right)+\sum {\underline{X}}_{j}^{2}n_{j}-{\left(\sum {\underline{X}}_{j}n_{j}\right)}^{2}/\sum n_{j}).$$

The within‐groups pooled standard deviation is then computed using the standard formula, equation (5) in Wilson (2015), where *T* denotes treated and C denotes control:

$$s_{pooled}=\surd \left(\left(s_{T}^{2}\left(n_{T}-1\right)+s_{C}^{2}\left(n_{C}-1\right)\right)/(n_{T}+n_{C}-2)\right).$$

Moskowitz (1981) further provided results separately by two subscales for two outcome measures, Self‐esteem and Locus of control. We conducted the data synthesis using a synthetic effect size (the average) of these two outcome measures to avoid dependence between effect sizes.

We provided a graphical display (forest plot) of effect sizes. Graphical displays for meta‐analysis performed on ratio scales sometimes use a log scale, as the confidence intervals then appear symmetric. This is however not the case for the software Revman 5, which we used in this review.

#### 4.3.10 Subgroup analysis and investigation of heterogeneity

We planned to investigate the following factors with the aim of explaining observed heterogeneity: study‐level summaries of participant characteristics (e.g., studies considering a specific gender, age or socioeconomic level or studies where separate effects for girls/boys, primary school/secondary school or low/high socioeconomic status are available) and quality of the service learning programme according to the standards as outlined in Section *The intervention*.

There were, however, insufficient studies for moderator analysis to be performed.

#### 4.3.11 Sensitivity analysis

There were not enough studies to evaluate whether the pooled effect sizes were robust across components of risk of bias. Sensitivity analysis was only used to examine the impact of the cluster correction.

## 5 RESULTS

### 5.1 Description of studies

#### 5.1.1 Results of the search

We summarised the search results in a flow chart in Figure 1. The total number of potential relevant studies was 13,719 after excluding duplicates (database: 12,324, grey, hand search, snowballing and other resources: 1,395). We screened all studies based on title and abstract; 13,134 were excluded for not fulfilling the screening criteria, six studies were unobtainable despite efforts to locate them through libraries and searches on the Internet (they are listed in Table 1) and 579 studies were ordered, retrieved, and screened in full text. Of these, 542 did not fulfil the screening criteria and were excluded. We included a total of 37 studies in the review. The references are listed in section *References to included studies*.

**Table 1:** Unobtainable studies

Author	Title	Source	Year
Krug J. L.	Select changes in high school students' self‐esteem and attitudes towards their school and community by their participation in service learning activities at a Rocky Mountain high school	Dissertation, University of Colorado at Boulder	1991
Papponi P.	The effect of remediation/enrichment, character education, and service learning on secondary students' self‐concept and academic achievement	Dissertation, The University of New Mexico	1999
Kinsley C.W.	Community service learning as a pedagogy.	Equity & Excellence in Education	1993
Malvin J. and Others	Evaluation of Two Alternatives Programs for Junior High School Students.	Pacific Inst for Research; Evaluation	1982
Pandina R. J., Johnson V. L. & Barr S. L.	Peer Group Connection: A peer‐led program targeting the transition into high school	Handbook of adolescent drug use prevention: Research, intervention strategies, and practice.	2015
Westrick J. M.	The influence of service‐learning on the development of intercultural sensitivity: A case of an international school in Hong Kong	Globalising minds: Rhetoric and realities in international schools.	2014

#### 5.1.2 Included studies

The search and screening resulted in a final selection of 37 studies, which met the inclusion criteria for this review. The 37 studies analysed 30 different populations. Only 10 studies (analysing nine different populations) could be used in the data synthesis. Eighteen studies were judged Critical risk of bias for either the confounding item (16), for the Selection bias data item (7) or for the Selection of Reported Results item (1) (see supplementary documents for the detailed risk of bias assessments are available here: https://osf.io/v8ceq/). Several of the studies were rated Critical risk of bias on one or more of the risk of bias items. In accordance with the protocol, we excluded studies rated Critical risk of bias on any of the risk of bias items from the data synthesis on the basis that they would be more likely to mislead than inform. Five studies did not provide enough information enabling us to calculate an effect size and standard error, or did not provide results in a form enabling us to use it in the data synthesis. Further, one study was a Brief Issue and did not provide enough information to assess risk of bias and in addition only a subset of selected outcomes were reported. Attempt to locate the full evaluation was not successful.

Finally, of the studies that could be used in the data synthesis, two clusters of studies used the same data sets and reported on the same outcome(s), thus in addition three studies were not used in the data synthesis, see below.

Three studies analysed the same data set (Melchior, 1995, 1998, 1999). We could not extract enough information to calculate effect sizes from Melchior (1999) and the Melchior (1998) study contained more information than the Melchior (1995) study, thus we used the Melchior (1998) study in the data synthesis.

Four studies analysed the same cluster randomised trial in Florida: Walsh‐Buhi (2016), Daley & Buhi (2015), Daley (2019) and Debate (2018). The trial included two cohorts, and two follow‐up times. The four studies varied on the cohorts, follow‐up times and outcomes they reported on. Both Walsh‐Buhi (2016) and Daley (2019) were used in the data synthesis as they reported outcomes at different time points. We did not use Daley and Buhi (2015) as the results were reported as probability differences and the same outcomes at the same time points were reported as odds ratios in Daley (2019). The study Debate (2018) was not used in the data synthesis as it was not possible to calculate an effect size from the information provided. In addition, one study, Francis (2016), was a summary on five trials of which four were included in this review (including the Florida trial). The individual studies reporting on these four trials provided more information than the summary and thus the summary study was not used in the data synthesis.

Two studies, Curtin (2008, 2011), reported on the same data set, but both studies were rated Critical risk of bias and was not used in the data synthesis.

In Table 2 we show the total number of studies, that met the inclusion criteria for this review. The first column shows the total number of studies grouped by country of origin. The second column shows the number of these studies that did not provide enough data to calculate an effect estimate. The third column gives the number of studies that were coded with Critical risk of bias. The fourth column gives the number of studies that were excluded from the data synthesis due to using the same data sets. The last column gives the total number of studies used in the data synthesis.

**Table 2:** Number of included studies by country

		Reduction due to			
Country	Total	Missing data	Critical risk of bias	Used same data sets	Used in data synthesis
USA	36	5	18	3	10
Nigeria	1	1			0

*Note*: The reduction due to Critical risk of bias preceded the reduction due to using same data set.

Eighteen studies could not be used in the data synthesis as all reported outcomes were judged to have a critical risk of bias.

Six studies did not provide enough information enabling us to calculate an effect size and standard error or did not provide results in a form enabling us to use it in the data synthesis, and finally three studies were not used due to reporting on the same outcomes from the same populations. We listed all studies in Table 3 along with the reason why the study was not used in the data synthesis.

**Table 3:** Characteristics of included studies

Study	Country	Outcome	Used in data synthesis/reason not used
Ajitoni 2015	Nigeria	Environmental knowledge	Cannot calculate effect size
Baumann 2014	USA		Not enough information to assess anything.
Billig 2008	USA	Attendance rates, in‐school suspensions, out‐of‐school suspensions, and serious incidents.	Rated Critical risk of bias
Curtin 2008	USA	Social competence, and academic achievement	Rated Critical risk of bias
Curtin 2011	USA	See Curtin 2008	Same data as Curtin 2008, rated Critical risk of bias
Daley & Buhi 2015	USA	Ever having been pregnant or gotten someone pregnant	Do not use the results reported in this study as these outcomes are reported as OR in Daley et al., 2019 at both follow up times (although only for cohort 1) which we use.
Daley 2019	USA	Recent risky sex, ever having been pregnant or gotten someone pregnant	Same trial as Daley & Buhi (2015). Used in data synthesis
DeBate 2018	USA	Competence, Confidence, Connection, Character, and Caring)	Same trial as Daley & Buhi (2015). Not possible to calculate effect size
Dones 1999	USA	Locus of control	Used in data synthesis
Elliott 2015	USA	Math Score, Math Identity, Math Self‐Efficacy, Science Self‐Efficacy, Science Identity and School Engagement	Rated Critical risk of bias
Emerson 2011	USA	Attitudes towards people with a variety of disabilities (ACL), children's behavioural intentions towards children with disabilities (FAS), School Autonomy and Influence, efficacy	Rated Critical risk of bias
Fraley 2015	USA	GPAs, incidents of discipline, attendance, and dropout rate	Rated Critical risk of bias
Francis 2015	USA	Engagement in unprotected sex	Used in data synthesis
Francis 2016	USA		Summary of five trials reported on elsewhere, not used
Giacalone 2004	USA	Self‐esteem	Used in data synthesis
Hanna 2014	USA	Emotional intelligence and sub scales	Rated Critical risk of bias
Jaffe 1998	USA	Oral reading	Rated Critical risk of bias
Kuhns 2011	USA	Self‐concept	Rated Critical risk of bias
Leming 2001	USA	Self‐esteem, sense of responsibility	Cannot calculate effect size and too little information to assess ROB
McFarland 2015	USA	Graduation	Rated Critical risk of bias
Mclouglin 2009	USA	Psychosocial development, various measures	Rated Critical risk of bias
McNamara 2000	USA	Absences, tardies and grade point average	Rated Critical risk of bias
Melchior 1995	USA	Same as Melchior, 1998	Same as Melchior, 1998, not used in data synthesis
Melchior 1998	USA	Personal and Social Responsibility, educational development and academic performance, personal and social development, Consumed any Alcohol in Past 30 Days; Used Illegal Drugs in Past 30 Days; Arrested in Past 6 Months; Ever Pregnant or Made Someone Pregnant; Fought, Hurt Someone, or Used Weapon in Last 6 Months)	Used in data synthesis
Melchior 1999	USA	Same as Melchior, 1998	Same data as Melchior, 1998 and cannot calculate effects size
Miller 2009	USA	Political self‐efficacy, Community presence self‐efficacy, Community service self‐efficacy	Rated Critical risk of bias.
Moskowitz 1981	USA	GPA, unexcused absences, academic self‐esteem, social self‐esteem, locus of control (success and failure) and Nondrug problems	Used in data synthesis
O'Donnell 2002	USA	Ever been/have made anybody pregnant	Cannot calculate ES and impossible to rate risk of bias as too little information is provided
Perry 1998	USA	School belonging, self‐esteem	Rated Critical risk of bias
Philliper 2015	USA	Ever been/caused someone to be pregnant and risky sex	Used in data synthesis
Philliper 2016	USA	Risky sex (lack of recent birth control use)	Used in data synthesis
Rossi 2002	USA	Social and Personal Responsibility	Rated Critical risk of bias
Santmire 1999	USA	Student achievement	Used in data synthesis
Scales 2000	USA	Social responsibility, Personal development opportunities, Commitment to classwork, Engagement with school, Intellectual achievement responsibility, GPA average and conduct average	Rated Critical risk of bias
Walsh‐Buhi 2016	USA	Risky sex (without condom).	Same trial as Daley & Buhi (2015). Used in data synthesis
Welkowitz 2001	USA	Self‐control, affective development, Effortful Engagement, Effortful Disengagement, Involuntary Engagement, and Involuntary Disengagement, Grades, attendance, discipline referrals	Rated Critical risk of bias
Williams 1997	USA	Attitudes towards school, towards helping others, and towards future life goals; school attendance, school disciplinary offenses	Rated Critical risk of bias

The main characteristics of the 10 studies (analysing nine populations) used in the data synthesis are shown in Table 4. Note that the participants in the trial reported in multiple studies only appears once in Table 4.

**Table 4:** Characteristics of studies used in data synthesis

*Characteristic (number of studies reporting)*	
Year of intervention (7)	Average (SD) 2007 (10)
	Range 1980–2013
Number of participants, treated (9)	Average (SD) 937 (1306)
	Range 18–3556
Number of participants, control (9)	Average (SD): 927 (1398)
	Range 20–3395
Percent female (7)	Average (SD) 54 (6)
Mean age (5)	Average (SD) 13 (2)
	Range 9–15
Percent white (7)	Average (SD) 56 (31)
	Range 12–96
*Programme features*	
Linking to curriculum (7)	Yes: 7 studies
	No: 0 studies
Having a Youth voice (6)	Yes: 4 studies
	No: 2 studies
Community involvement (5)	Yes: 5 studies
	No: 0 studies
Reflection (8)	Yes: 8 studies
	No: 0 studies
Duration of intervention in months (8)	Average (SD) 7 (3)
	Range 1‐9
Hours of planned community service per week (6)	Average (SD) 1.3 (1.4)
	Range 0.5–4
Any implementation problems described	Yes 8
	No 1

Note that the participants in the trial reported in multiple studies (Daley, 2019; Walsh‐Buhi, 2016) only appears once.

^a^ Effect size is Hedges *g* and a positive effect favours the treated.

^b^ effect size is probability difference and negative effect favours the treated.

The timespan in which included studies were carried out is 33 years, from 1980 to 2013 and on average the intervention year was 2007 (not reported in two studies). The average number of participants in service learning analysed was 937, ranging from 18 to 3556 and the average number of controls was 927, ranging from 20 to 3395. Not all studies reported an average age of the participants but among those that did the average was 13 years ranging from 9 to 15 years (not reported in four studies). Likewise, a limited number of studies reported other characteristics of study participants. On average females constituted a little more than half of service learning participants, 54% (not reported in two studies). Ethnicity of service learning participants was reported in seven studies and the average percent of white students was 56% with great variation, ranging from 12% to 96%.

Concerning quality of the service learning programmes according to the five standards we wanted to focus on, seven studies reported linking programmes to academic and program curriculum or objectives and none reported not following this standard; four studies reported incorporating youth voice whereas two studies reported not incorporating youth voice; involving community partners was reported in five studies and eight studies reported providing opportunities for reflection and one study did not report on this standard. Lastly, concerning duration and intensity, the average duration of service learning was seven months (not reported in one study) but with great variation, ranging from 1 month to nine months. The hours of planned community service was on average 1.3 per week, ranging from 0.5 h per week to 4 h per week. Although the actual number of community service hours was not reported in the majority of studies (two studies reported the actual average number received), it was probably lower than the planned as all but one study reported implementation problems.

#### 5.1.3 Excluded studies

In addition to the 37 studies that met the inclusion criteria for this review, 60 studies at first sight appeared relevant but did not meet our criteria for inclusion. The studies and reasons for exclusion are given in Table 5. More than a third (24 studies) were excluded as they compared one single unit (school or class) to another (or several other units).

**Table 5:** Excluded studies

Study	Reason for exclusion
Akers 2008	One school is treated and another is control
Allen 1990	This study was embedded within a larger evaluation that used a quasiexperimental design involving Teen Outreach students and a comparison group of students closely matched on various background characteristics (Philliber et al., 1989). Some are after‐school implementations.
Allen 1991	A description of the program and some features associated with its succes
Allen 1994	Uses a subsample from a larger study where an unknown number of program recruits students to after‐school implementations
Allen 1997	Probably most of the programs are after‐school programs
Allen 2001	This study thus utilised data collected over a 4‐year period across over 60 sites nationwide, years not reported. Some participated as an after‐school activity. Exclude as not all receive the program in school
Arrington 2010	One class is treated and another class is control
Benigni 2006	No relevant outcomes
Billig 2005	No relevant outcomes
Bull 2015	A study of a SL program with an add on and control receives only the SL program
Bull 2016	A study of a SL program with an add on and control receives only the SL program
Campbell 2000	Voluntary work in general and not service learning
Cardona 2013	12 different science classrooms and 267 eighth grade students, 6 classrooms in each condition but only one teacher in each condition teaching 6 classes
Chun 2009	No relevant outcomes (non‐standardised and/or researcher developed)
Clark 2017	One classroom/teacher is treated (p. 63) unclear concerning control but probably another classroom/teacher
Cofer 1996	Three different projects each analysed separately with one class treated and one class control
Condon 2018	No relevant outcomes, all are non standardised
Dallago 2009	Outcomes collected are: self‐efficacy, empowerment, civic responsibility towards the neighbourhood, neighbourhood attachment, i.e. the first two are relevant for this review but the authors only report the significant results which is civic responsibility, i.e. no relevant results are reported.
Dawn 2008	Not SL, an after‐school community learning service activity and comparison is one class assessed the following year
Dean 2002	No relevant outcomes, none are standardised
Dinan 2005	Not an individual level analysis but a school level analysis, compares public high schools with and without state‐recognised service‐learning programs
Flores 2018	Only outcome is Civic engagement measured by the Civic Responsibility Survey for K‐12 Students Engaged in Service
Furco 1997	No relevant outcomes (non‐standardised and researcher developed)
Galati 2004	Students from one county (one school) compared to students from two other counties (two schools)
Green‐Tucker 2016	Not service learning, half of a co‐thaught math/family/science and technology class apparantly go to Ghana to build water wells
Gullo 2012	One unit (teacher or time) compared to one unit (teacher or time)
Hecht 1995	Only outcome is caring, not measured by a standardised instrument
Hecht 1997	Discusses another impact study but do not report results
Henderson 2007	Not SL and no relevant outcomes
Henness 2001	Control and comparison groups were developed on the basis of whether service‐learning projects addressed high or low community priorities.
Howard 2006	No control group (only pre not post) and no relevant outcome (hours watching TV)
Kamm 2007	One school is the treated school and another school is control 1 and a third school is control 3
Klassen 2012	Students in one classroom were treated and students in another classroom were control
Lakin 2006	Two classes treated and one class control
Lee 2007	No relevant outcomes
Levine 2016	Afterschool programme
Lomino 2003	Treated from the same school and controls from other schools
Marks 1994	No relevant outcomes and probably not service learning
Martin 2006	Report on a survey which includes homeschooled students in an unknown number. It is a nationally representative survey of 3,123 U.S. residents ages 18‐28, asking in retrospect about service learning participation in school.
McBride 2014	One school compared to another school
McBride 2016	One school is treated and another is comparison
Merle 1998	Treated from two schools in one particular year and comparison from one of these schools in the two previous years, thus one unit compared to another unit
Milton 2011	Treatment in one middle school and comparison from other middle schools (although all at the time of data collection attend the same high school)
Moss 2010	One school is treated and another is comparison
Ocal 2016	Treated in one school and control in the other school
O'Donnell 1999	One school is treated and another is control.
O'Donnell 1999a	One school is treated and another control
Palkowski 2006	Compares to one control classroom
Quinn 1995	One classroom treated and one classroom control
Richards 2013	One school is treated and two other schools are comparison
Roberts 1997	Refers to Tables 1 and 2 for results but they are not displayed in the article and cannot find them anywhere else and cannot find an e‐mail adress for the authors (one of them apparantly dead)
Robinson 2016	Not a school intervention
Schneller 2008	One class/one teacher in each of the two groups
Seshadri 2015	Comparison also receives SL and the study is effectively testing the additive effect of TOP
Stewart 2013	One classroom treated and one classroom control
Trager 2011	Analysis on district level: district dropout rate was the dependent variable and whether the school district received a Learn and Serve America grant as the independent variable of interest
Waldstein 2001	No standardised outcomes reported, use a modification of (perhaps) validated instrument
Wang 1998	Three different projects each analysed separately with one class treated and one class control
Whitelaw 2004	No relevant outcomes
Yamauchi 2006	No relevant outcomes

### 5.2 Risk of bias in included studies

The risk of bias coding for each of the 37 studies is shown in a supplementary document (available here: https://osf.io/v8ceq/).

Fourteen studies reported on 10 randomised trials, two individually randomised trials and eight cluster randomised trials (reported in 12 studies). Four studies reported on the same CRCT, which included two cohorts, and two follow‐up times. The four studies varied on the cohorts, follow‐up times and outcomes they reported on, therefore they were all assessed for risk of bias. One study was a summary of five trials of which four were included in this review and this summary was not assessed for risk of bias.

Table 6 shows a summary of the risk of bias associated with the randomised studies.

**Table 6:** Risk of bias randomised studies

Item	Low risk of bias	Some concerns	High risk of bias	Unclear
Overall judgement	0	1	10	2
Randomisation Process	2	4	4	3
Deviations from intervention	1	10	0	2
Missing Outcome Data	2	6	3	2
Measurement of Outcome	1	9	1	2
Selection of Reported Results	0	8	4	1

Three of the studies did not report the method of randomisation nor was any baseline imbalances shown or discussed. We rated these three studies Unclear on the Randomisation Process item. Two studies reported an appropriate randomisation method and baseline balance on the pre‐specified confounders and were rated Low risk of bias. Another four studies had some issues and were rated Some concerns and the remaining four studies were rated High risk of bias. On the Deviations from intervention item, the majority of studies, 10 studies, had some issues and were rated Some concerns, one was rated Low risk of bias and the two did not provide any information and were rated Unclear.

Concerning missing outcome data, two studies had no issues, and we rated them Low risk of bias, two studies did not report information concerning missing data and were rated Unclear, and six respectively three studies were rated Some concerns and High risk of bias. All but one study had some issues on the Measurement of Outcome item, we rated nine Some concerns, one High risk of bias, and two studies did not provide enough information and were rated Unclear. We rated no study Low risk of bias on the Selection of Reported Results item, all but one were rated either Some concerns (eight studies) or High risk of bias (four studies) and the last was rated Unclear. Overall, none of the studies were rated Low risk of bias, the majority were rated High risk of bias (ten studies), one was rated Some concerns and two studies provided insufficient information and were rated Unclear overall.

The remaining 23 studies used non‐randomised designs, three studies (Melchior, 1995, 1998, 1999) used the same data set and modelling strategy so only one of them was risk of bias assessed. Table 7 shows a summary of the risk of bias associated with the non‐randomised studies. As stated in the protocol, we stopped the assessment of a non‐randomised study outcome when it was rated ‘Critical', therefore not all studies are rated on all domains.

**Table 7:** Risk of bias non‐randomised studies

Item	Low risk of bias	Moderate risk of bias	Serious risk of bias	Critical risk of bias	No information	Not rated
Overall judgement	0	0	1	18	2	0
Confounding bias	0	0	3	16	2	0
Selection bias	0	1	4	7	2	7
Classification bias	1	0	1	0	3	16
Deviation bias	2	0	1	0	2	16
Missing data	1	2	0	0	2	16
Measurement of Outcome	0	3	0	0	2	16
Selection of Reported Results	2	0	0	1	2	16

Eighteen of the non‐randomised studies were rated Critical risk of bias on the Overall judgement item corresponding to a risk of bias so high that the findings should not be considered in the data synthesis. The overall Critical risk of bias rating was mainly due to issues on the Confounding bias item; 16 were rated Critical risk of bias on this item; that is, they failed to establish a comparison group that was balanced on important confounders and further only a few controlled for any confounders. One study was rated Critical risk of bias overall due to a rating of Critical risk of bias on the Selection of Reported Results item. The remaining study rated Critical risk of bias on the Overall judgement item was rated Serious risk of bias on the Confounding bias and Selection bias items which lead to an Overall judgement rating of Critical risk of bias.

Two studies were rated Unclear overall as only very few, if any, of the domains in the risk of bias tool could be assessed due to very limited information provided. We excluded these two studies from the meta‐analysis. One study was rated Serious risk of bias overall and was used in the data synthesis.

### 5.3 Effects of interventions

Ten studies (analysing nine different populations) permitted calculation of an effect size and standard error and were not rated Critical risk of bias. A large variety of different outcomes were reported in the studies. To carry out a meta‐analysis, every study must have a comparable effect size. We synthesise effects separately by type of outcome (conceptual outcomes as outlined in section ‘Types of outcomes measures') and time point (end of intervention and follow up). Unfortunately each type of outcome was only reported in a small subset of studies (in many cases in only one single study). Thus, each meta analysis contains a very small number of effect sizes, at most three.

All continuous outcomes (effect sizes measured as Hedges g) were coded such that a larger effect size indicated better outcomes for the treated group. All binary outcomes (reported either as odds ratio or probability difference) were coded such that a smaller effect size indicated better outcomes for the treated group.

#### 5.3.1 Academic success post intervention

##### Student achievement

Three studies reported students' general GPA.

Two of the reported results indicated a positive effect favouring the treated and one indicated a negative effect favouring the comparison; none of the study‐level effects were statistically significant.

**Figure 2:**
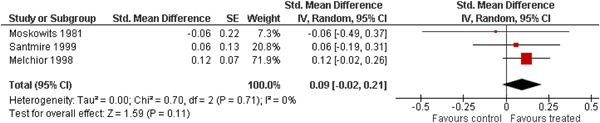
(Analysis 1.1) Forest plot of comparison: 1 Academic success, outcome: 1.1 Grade point average

The weighted average was positive and statistically nonsignificant. The random effects weighted standardised mean difference was 0.09 (95% CI −0.02 to 0.21). Although the *p* value of the *Q*‐statistic is notoriously underpowered to detect heterogeneity in small meta‐analyses, the estimated *τ* ^2^ is 0.00 and *I* ^2^ is 0%, implying that heterogeneity among these three studies was not present. The forest plot is displayed in Figure 2.

Two studies reported overall test results in reading.

One of the reported results indicated a positive effect favouring the treated and one indicated a negative effect favouring the comparison; none of the study‐level effects were statistically significant.

**Figure 3:**
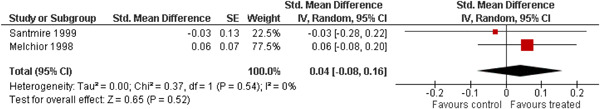
(Analysis 1.2) Forest plot of comparison: 1 Academic success, outcome: 1.2 Reading

The weighted average was positive and statistically nonsignificant. The random effects weighted standardised mean difference was 0.04 (95% CI −0.08 to 0.16). The estimated *τ* ^2^ was 0.00 and *I* ^2^ was 0%. The forest plot is displayed in Figure 3.

Two studies reported overall test results in math.

Both reported results indicated a positive effect favouring the treated; one of the study‐level effects was statistically significant and one was not statistically significant.

**Figure 4:**
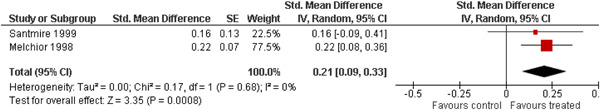
(Analysis 1.3) Forest plot of comparison: 1 Academic success, outcome: 1.3 Math

The weighted average was positive and statistically significant. The random effects weighted standardised mean difference was 0.21 (95% CI: 0.09 to 0.33). The estimated *τ* ^2^ was 0.00 and *I* ^2^ was 0%. The forest plot is displayed in Figure 4.

One study in addition to the overall math and reading test results, reported Social studies grades and Science grades and another study reported on a number of reading and math subscales in addition to the overall reading and math test results. We report the effect size in Table 8.

**Table 8:** Other outcomes

Study	Measure	Outcome	Effect size [95% CI]
*Academic success*			
Melchior (1998)	School records	Social studies grade^a^	0.16 [0.02, 0.30]
Melchior (1998)	School records	Science grade^a^	0.15 [0.01, 0.29]
Melchior (1998)	School records	Overall/School GPA (including electives, other courses)^a^	0.10 [−0.04, 0.24]
Santmire (1999)	Metropolitan Achievement Test (MAT)	Math Process^a^	0.21 [−0.04, 0.46]
Santmire (1999)	Metropolitan Achievement Test (MAT)	Math Concepts^a^	0.17 [−0.08, 0.42]
Santmire (1999)	Metropolitan Achievement Test (MAT)	Vocabulary^a^	0.01 [−0.24, 0.26]
Santmire (1999)	Metropolitan Achievement Test (MAT)	Reading comp.	−0.03 [−0.28, 0.22]
Santmire (1999)	Metropolitan Achievement Test (MAT)	Language^a^	0.12 [−0.13, 0.37]
Melchior (1998)	School records	Fail 1 or more courses^b^	−0.04 [−0.08, −0.00]
*Personal and social skills*			
Melchior (1998)	Personal and social responsibility (Search Institute scale: range 5–25):	Social welfare subscale^a^	0.18 [0.06, 0.30]
Melchior (1998)	Psychosocial maturity (Greenberger scale: range 1–4):	Communication Skills subscale^a^	−0.02 [−0.14, 0.10]
Melchior (1998)	Psychosocial maturity (Greenberger scale: range 1–4):	Work Orientation subscale^a^	0.06 [−0.06, 0.18]
Melchior (1998)	Connell scale	School engagement (Research/Assessment Package for Schools (RAPS))^a^	0.24 [0.12, 0.36]
*Risk behaviour*			
Melchior (1998)	Involvement with Risk Behavior (Search Institute, Profiles of Student Life)	Consumed any alcohol in last 30 days^b^	−0.02 [−0.08, 0.04]
Melchior (1998)	Involvement with Risk Behavior (Search Institute, Profiles of Student Life)	Used illegal drugs in last 30 days^b^	−0.01 [−0.05, 0.03]
Melchior (1998)	Involvement with Risk Behavior (Search Institute, Profiles of Student Life)	Arrested in last 6 months^b^	−0.00 [−0.04, 0.04]
Melchior (1998)	Involvement with Risk Behavior (Search Institute, Profiles of Student Life)	Fought, hurt, or used weapon in last 6 months^b^	−0.05 [−0.11, 0.01]
Melchior (1998)	Involvement with Risk Behavior (Search Institute, Profiles of Student Life)	Ever been pregnant or made someone pregnant^b^	−0.03 [−0.07, 0.01]
Melchior (1998)	School records	Suspended last year (days)^a^	0.03 [−0.15, 0.21]
Melchior (1998)	Involvement with Risk Behavior (Search Institute, Profiles of Student Life)	Total number of risk behaviours^a^	0.10 [−0.06, 0.26]
Moskowitz (1981)	School records	Nondrug problems^b^	0.04 [−0.39, 0.47]

^a^ Effect size is Hedges *g* and a positive effect favoures the treated.

^b^ Effect size is probability difference and negative effect favoures the treated.

##### Attendance

Two studies reported days absent from school.

Both reported results indicated a positive effect favouring the treated; none of the study‐level effects were statistically significant.

**Figure 5:**
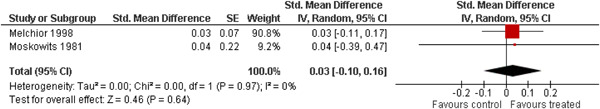
(Analysis 1.4) Forest plot of comparison: 1 Academic success, outcome: 1.4 Absences

The weighted average was positive and statistically nonsignificant. The random effects weighted standardised mean difference was 0.03 (95% CI: −0.10 to 0.16). The estimated *τ* ^2^ was 0.00 and *I* ^2^ was 0%. The forest plot is displayed in Figure 5.

##### Drop out, NEET and other outcomes

None of the studies reported on drop out or NEET status. One study reported on failure of courses. The effect size is reported in Table 8.

#### 5.3.2 Personal and social skills post intervention

Two studies reported on comparable self‐esteem measures.

Both reported results indicated a positive effect favouring the treated; none of the study‐level effects were statistically significant.

**Figure 6:**
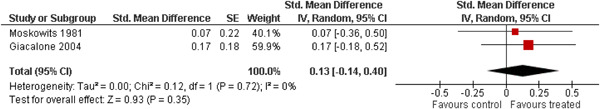
(Analysis 2.1) Forest plot of comparison: 2 Personal and social skills, outcome: 2.1 Self‐esteem

The weighted average was positive and statistically nonsignificant. The random effects weighted standardised mean difference was 0.13 (95% CI: −0.14 to 0.40). The estimated *τ* ^2^ was 0.00 and *I* ^2^ was 0%. The forest plot is displayed in Figure 6.

**Figure 7:**
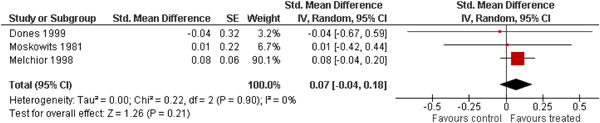
(Analysis 2.2) Forest plot of comparison: 2 Personal and social skills, outcome: 2.2 Locus of control

Three studies reported on measures of locus of control. None of the study‐level effects were statistically significant, and the weighted average was positive and statistically nonsignificant. The random effects weighted standardised mean difference was 0.07 (95% CI: −0.04 to 0.18). The estimated *τ* ^2^ was 0.00 and *I* ^2^ was 0%. The forest plot is displayed in Figure 7.

In addition, three studies reported on a number of other personal and social skills outcomes (measured by a variety of different standardised scales). These could however, not be meta analysed as each outcome was reported in only one study. The effect size is reported in Table 8.

#### 5.3.3 Risk behaviour postintervention

##### Pregnancy

Two studies reported on a pregnancy outcome (Have you ever been/caused someone to be pregnant) measured as an odds ratio.

**Figure 8:**
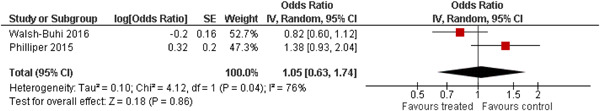
(Analysis 3.1) Forest plot of comparison: 3 Risk behaviour, outcome: 3.2 Ever been/made someone pregnant

An odds ratio less than 1 indicates that the treated, that is, the participants in service learning, is favoured. That is, the odds of having been or caused someone to become pregnant is lower for participants in service learning. One of the reported results indicated an effect favouring the treated and one indicated an effect favouring the comparison. The weighted average favoured the comparison and was statistically nonsignificant. The random effects weighted mean odds ratio was 1.05 (95% CI: 0.63 to 1.74). The forest plot is displayed in Figure 8. There was some heterogeneity between the studies; the estimated *τ* ^2^ was 0.10, *Q* = 4.12, *df* = 1 and *I* ^2^ was 76% as displayed in Figure 8.

In addition, one study measured this outcome as a probability difference (reported as the model result from a linear regression, hence it could not be transformed to an odds ratio). The effect size is reported in Table 8.

##### Risky sex

Three studies reported on a risky sex outcome (Engagement in unprotected sex) measured as an odds ratio.

Two of the reported results indicated an effect favouring the treated and one indicated an effect favouring the comparison; none of the study‐level effects were statistically significant.

**Figure 9:**
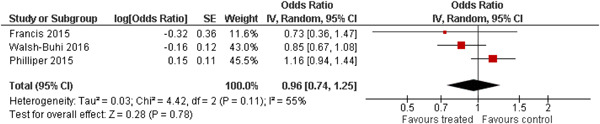
(Analysis 3.2) Forest plot of comparison: 3 Risk behaviour, outcome: 3.1 Risky sex

The weighted average favoured the treated and was statistically nonsignificant. The random effects weighted mean odds ratio was 0.96 (95% CI: 0.74 to 1.25). There was some heterogeneity between the studies; the estimated *τ* ^2^ was 0.03, *Q* = 4.42, *df* = 2 and *I* ^2^ was 55%. The forest plot is displayed in Figure 9.

##### Other risk behaviour

A number of other risk behaviours were reported in two studies but could not be meta analysed as each outcome was reported in only one study, see Table 8 for the effect sizes.

#### 5.3.4 Academic success at follow up

None of the studies reported academic success outcomes at follow up (one study actually did, but follow‐up outcomes were rated Critical risk of bias, see the supplementary document here: https://osf.io/v8ceq/).

#### 5.3.5 Personal and social skills at follow up

None of the studies reported personal and social skills outcomes at follow up (one study actually did, but follow‐up outcomes were rated Critical risk of bias, see the supplementary document here: https://osf.io/v8ceq/).

#### 5.3.6 Risk behaviour at follow up

##### Pregnancy

Two studies reported on the pregnancy outcome (Have you ever been/caused someone to be pregnant) measured as an odds ratio approximately one year after the intervention.

**Figure 10:**
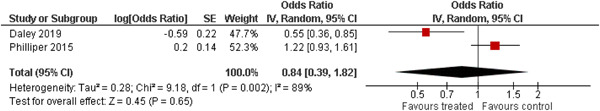
(Analysis 3.4) Forest plot of comparison: 3 Risk behaviour, outcome: 3.5 Ever been/made someone pregnant Follow up

One of the reported results indicated an effect favouring the treated and one indicated an effect favouring the comparison. The weighted average favoured the treated and was statistically nonsignificant. The random effects weighted mean odds ratio was 0.84 (95% CI: 0.39 to 1.82). There was heterogeneity between the studies; the estimated *τ* ^2^ was 0.28, *Q* = 9.18, *df *= 1 and *I* ^2^ was 89% as displayed in Figure 10.

##### Risky sex

Three studies reported on a risky sex outcome (Engagement in unprotected sex) measured as an odds ratio approximately one year after the intervention.

**Figure 11:**
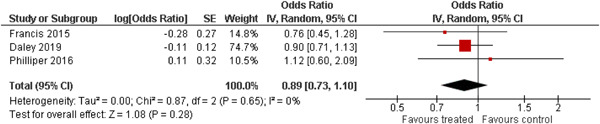
(Analysis 3.5) Forest plot of comparison: 3 Risk behaviour, outcome: 3.4 Risky sex Follow up

Two of the reported results indicated an effect favouring the treated and one indicated an effect favouring the comparison; none of the study‐level effects were statistically significant. The weighted average favoured the treated and was statistically nonsignificant. The random effects weighted mean odds ratio was 0.89 (95% CI: 0.73 to 1.105). We found no heterogeneity between the studies; the estimated *τ* ^2^ was 0.00, and *I* ^2^ was 0%. The forest plot is displayed in Figure 11.

##### Other risk behaviour

None of the studies reported other risk behaviour outcomes at follow up (one study actually did, but follow‐up outcomes were rated Critical risk of bias, see the supplementary document here: https://osf.io/v8ceq/).

#### 5.3.7 Sensitivity

Two studies used in the meta analyses needed correction for treatment given in clusters. One study was a cluster randomised trial (Giacalone, 2004) and the other was an individualised randomised trial (Santmire, 1999).

**Figure 12:**
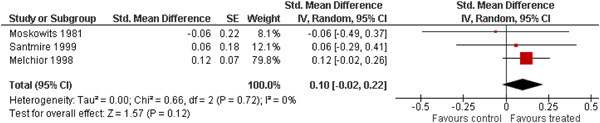
(Analysis 4.2) Forest plot of comparison: 4 Sensitivity, outcome: 4.2 Sensitivity Grade point average

**Figure 13:**
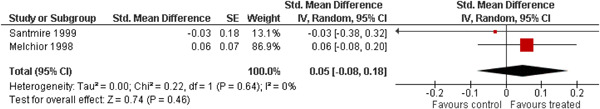
(Analysis 4.5) Forest plot of comparison: 4 Sensitivity, outcome: 4.5 Sensitivity Reading

**Figure 14:**
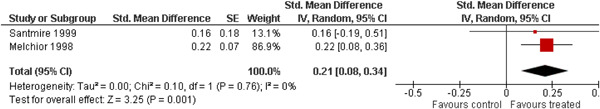
(Analysis 4.6) Forest plot of comparison: 4 Sensitivity, outcome: 4.6 Sensitivity Math

**Figure 15:**
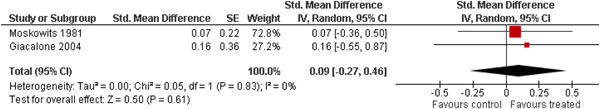
(Analysis 4.7) Forest plot of comparison: 4 Sensitivity, outcome: 4.7 Sensitivity Self‐esteem

Although adjusting for clustering decreased the individual effect sizes slightly and increases the standard errors, the average effect size estimates were virtually unchanged, and the conclusions did not change (Figures 12, 13, 14, 15).

Sensitivity analyses were further planned to evaluate whether the pooled effect sizes were robust across study design and components of methodological quality. However, there was no meta‐analysis in which the number of studies contributing data was sufficient for further sensitivity analysis to be meaningful (no meta‐analysis included more than three studies).

## 6 DISCUSSION

### 6.1 Summary of main results

Overall, there were too few studies included in any of the meta‐analyses in order for us to draw any conclusion concerning the effectiveness of service learning on student outcomes. At most, the results from three studies could be pooled in a single meta‐analysis. All the meta‐analyses showed a positive weighted average except the pregnancy outcome and none of them was statistically significant except the weighted average of the two studies reporting math test results.

### 6.2 Overall completeness and applicability of evidence

We included in total 10 studies (analysing nine different populations) in the data synthesis and of these a maximum of three studies reported the same outcome and could be used in a specific meta‐analysis. This number is lower than the number of studies (37) meeting the inclusion criteria. The reduction was caused by three different factors.

Six studies did not report effect estimates or provide data that would allow the calculation of an effect size. Eighteen studies were judged to have a Critical risk of bias and, in accordance with the protocol, we excluded these from the data synthesis on the basis that they would be more likely to mislead than inform. Finally, we could not use three studies as they reported on two clusters of studies using the same data sets and reporting on the same outcome(s) at the same time points.

If all the included studies had provided an effect estimate with lower risk of bias, the final list of useable studies in the data synthesis would have been larger, which again would have provided a more robust literature on which to base conclusions.

All studies used in the data synthesis were from the United States. A single study outside the United States was identified (from Nigeria) but did not provide data that would allow the calculation of an effect size. This narrow geographical coverage is a clear limitation of the review.

Follow‐up analyses were only possible for two risk behaviour outcomes and none of the other types of outcomes. This is also a clear limitation of the review.

It was not possible to examine the impact of the moderators nor sensitivity analyses for each outcome to check whether the obtained results were robust across study design and methodological quality.

### 6.3 Quality of the evidence

The majority of studies (23) used non‐randomised designs, and 14 studies reported on 10 randomised trials. Overall the risk of bias in the included studies was high. Among the non‐randomised studies only one study was not rated Critical risk of bias (in addition, two studies provided too little information to be rated). The level ‘Critical’ means: the study (outcome) is too problematic in this domain to provide any useful evidence on the effects of intervention, and it is excluded from the data synthesis.

None of the randomised trials were overall rated low risk of bias, one was assessed to have some concerns while the rest were of high risk of bias (in addition two studies provided too little information to be rated).

We examined the risk of bias using Cochranes revised risk of bias tool, RoB 2 (Higgins et al., 2019) for the randomised studies and using the model ROBINS–I, developed by members of the Cochrane Bias Methods Group and the Cochrane Non‐Randomised Studies Methods Group (Sterne et al., 2016a) for the non‐randomised studies.

The quality of the evidence in this review was enhanced by excluding studies assessed to be at critical risk of bias using the ROBINS–I tool from the data synthesis. We believe this process excluded those studies that are more likely to mislead than inform.

There was overall consistency in the direction and magnitude of effects and there was no heterogeneity between studies except in a few cases (the risk behaviour outcomes).

### 6.4 Potential biases in the review process

We performed a comprehensive electronic database search, combined with grey literature searching, and hand searching of key journals. All citations were screened in teams by two independent screeners from the review team (TPC, MCTM, FSB, and LMTD), and one review author (TF) assessed all included studies against inclusion criteria (the review team is listed in section Acknowledgements).

We believe that all the publicly available studies on the effect of service learning on students' academic success, personal and social skills and risk behaviour up to the censor date were identified during the review process. However, six references were not obtained in full text.

We were unable to comment on the possibility of publication bias as at most three studies was included in the same meta‐analysis. Thus, we cannot rule out that there are still some missing studies, which were not published or made public.

We believe that there are no other potential biases in the review process as two teams each with two members of the review team (TPC, MCTM, FSB, LMTD) independently coded the included studies. Any disagreements were resolved by discussion. Further, decisions about inclusion of studies were made by the two teams of each two members of the review team (TPC, MCTM, FSB, LMTD) and one review author (TF). Assessment of study quality and numeric data extraction was made by one review author (TF) and each study was checked by at least another review author (JD, NTD) and in addition in some cases by two members of the review team (TPC, MCTM).

### 6.5 Agreements and disagreements with other studies or reviews

The review by Celio et al. (2011) compared service learning interventions to control groups and is the only review we believe can be compared to our review. Celio et al. (2011) found 62 studies, of which 19 had participants from primary and secondary education only. Five outcome areas were analysed: attitudes towards self, attitudes towards school and learning, civic engagement, social skills, and academic achievement. Separate results for primary and secondary education (grades kindergarten to 12) was only shown for the overall effect, that is, the mean of the five outcomes attitudes towards self, attitudes towards school and learning, civic engagement, social skills, and academic achievement. The overall average effect (obtained from a random effects model) for these five measures combined was 0.20 for K‐12 students (95% CI: 0.08 to 0.31); higher than any of the measures analysed separately in our review except for math. It is, however, unclear how much each of the five measures contributed to the size of the combined single outcome effect size. From the 62 studies (including those not analysing college and beyond) a total of 380 effect sizes were extracted and used in their meta‐analyses. It is not reported how large a share of these effect sizes were from K‐12 studies.

The approach followed by Celio et al. (2011) differ from ours in two other important aspects, making it difficult to compare the results. First, contrary to our inclusion criteria Celio et al. (2011) did not require outcome measures to be reliable or valid; of the total 380 effect sizes included, 120 was coded as not ‘Use of reliable outcome measures’ and only 169 were coded as ‘Use of valid outcome measures’ (tab. 3 in Celio et al., 2011). The average effect size in studies that used reliable outcome measures was markedly smaller than in those that did not (0.23 compared to 0.41), whereas the effect size calculated based on valid outcomes was similar to the one based on not validated measures (0.27 compared to 0.30). However, Celio et al. did not report these subgroup analyses separately for K‐12 students and further they did not take into consideration that more than one outcome per study was included in this subgroup analysis (i.e., they did not take into account the statistical dependencies between the effect sizes).

Second, Celio et al. (2011) included all studies in their meta‐analyses whereas we excluded studies rated Critical in at least one domain of ROBINS‐I. RCTs and non‐randomised studies have very similar effect sizes in their analyses (0.31 compared to 0.30). However, as this subgroup analysis is not reported separately for K‐12 studies, it is difficult to say whether this result holds also in the subgroup of interest in our review.

## 7 AUTHORS' CONCLUSIONS

### 7.1 Implications for practice

The current landscape of research on service learning in primary and secondary general education (grades kindergarten to 12) shows that it has yet to be evaluated thoroughly. The evidence was inconclusive because too few studies reported results on the same type of outcome.

Furthermore, all the available evidence used in the data synthesis was USA‐based, and so the findings may not be generalisable to other settings and systems outside the United States. In fact, as the educational systems within the US differ between states and the studies examined service learning in different communities and settings, generalisations between contexts within the USA should also be made with care. However, it is important to point out that service learning is, in our view, potentially applicable in a wide range of contexts, and service learning interventions could be implemented in many more countries than those found in the studies we included. That is, the reason for the low number of studies from other countries may have less to do with institutional constraints and more to do with the tradition of quantitative educational research being stronger in the USA than elsewhere (see e.g., Dietrichson et al., 2020, 2021, for a similar pattern of USA‐dominance regarding interventions targeting students with academic difficulties).

### 7.2 Implications for research

In this review, we aimed to find evidence of the effectiveness of service learning on students' academic success, personal and social skills, and risk behaviour. However, the evidence was inconclusive. We found only few randomised controlled trials and the risk of bias in the included non‐randomised studies was very high leaving only one non‐randomised study to be meta‐analysed. The majority of the eight randomised trials available for meta‐analysis reported on a very limited number of outcomes; in particular few reported results on students' academic success even though the outcome was collected. Furthermore, the majority of studies used in the meta‐analyses reported implementation problems.

These considerations point to the need for more rigorously conducted studies reporting a larger number of outcomes.

It would be natural to consider conducting a large randomised controlled trial (or a series of large randomised trials) with specific allocation to implementation of high quality service learning as guided by the eight standards: (1) Meaningful service, (2) Link to curriculum, (3) Reflection, (4) Diversity, (5) Youth voice, (6) Community partnerships, (7) Progress monitoring and (8) Sufficient duration and intensity. Moreover, high‐quality service learning practice occurs when it is shaped and adapted to the particular community and student contexts and conditions. When setting up an experiment, the intervention should therefore allow for adaptations to the community and student context. For example, the particular community service project should not be required to be the same across sites, as such a requirement would violate the standards of meaningful service, youth voice, and partnerships. What is important is that students can self‐select into a service learning activity of their choosing, for the service learning effort to be considered of high quality. Students in the same class need not even participate in the same activity, teams of students within a class performing different activities of their own choice would meet the standards of high‐quality service learning.

These features of high‐quality service learning present some difficulties for the design and implementation of a high‐quality randomised trial. As chosen projects can be collaborations between students and to reduce the risk of spill‐over effects, the intervention should be assigned to clusters of students, not individual students. One could imagine a cluster‐randomised trial where either classes within schools are randomised to take up service learning in a particular course, or schools are randomised to offer service learning in particular grades, or whole school districts are randomised to implement service learning. Larger clusters decrease the risk of spill‐overs but may increase implementation difficulties. In this regard, there are examples of class (e.g., Schanzenbach, 2007), school (e.g., Gersten et al., 2015), and school district (e.g., Slavin et al., 2013) randomised trials in other areas of educational research, which we believe have yielded informative results and which much can be learnt from.

If schools can adapt and students self‐select into the service learning activities, then it will be more difficult to find suitable outcome measures than if the intervention was the same across sites. However, some measures used by the included studies in this review were both validated and broad enough to capture effects that would be interesting to examine for any service learning activity. Examples include school absences, self‐esteem, self‐efficacy, social skills, and locus of control. No meta analysed study used drop out or on‐time graduation but such measures would be interesting outcomes in any service learning intervention. Furthermore, while the service learning activity may differ, the subject in which service learning is implemented could be standardised across sites, possibly without decreasing the quality of the service learning experience. There are many validated tests available that could be used to examine effects on important student skills such as math and reading. By standardising for example state‐level tests that all students are expected to take (e.g., by using the percentile rank within states), such tests can be meaningfully compared also across tests. Although measures of broader skills may not capture effects on aspects inherent to the service learning activity, this trade off would be acceptable in our view.

Specific attention would also have to be paid to stringency in terms of conducting a well‐designed randomised trial with low risk of bias as well as ensuring that the sample sizes are large enough to enable sufficient power. The trial or trials should be designed, conducted, and reported according to methodological criteria for rigour to achieve high internal validity. For example, by following the criteria for risk of bias laid out in the RoB‐2 tool (Higgins et al., 2019). To achieve high external validity, schools and students should be sampled from differing contexts and outcomes should be measured with validated instruments. If possible, research designs that allow for the evaluation of both short and long‐term effects would be preferable.

Although we believe that implementing high‐quality randomised trials is possible, we want to acknowledge that it is challenging (in all areas of education). Supplementing randomised trials with high‐quality quasi‐experimental studies will therefore be important to learn more about the effects of service learning. For instance, no included study used a ‘natural experiment’ to estimate the effects of service learning. The variation of service learning mandates across both time and school districts in the United States (Education Commission of the States, 2014; Spring et al., 2008) suggests that students have been differentially exposed to service learning because of factors that may be unrelated to student and school characteristics. Similar differences across regions and time have been used in other areas of education (see Gopalan et al., 2020 for a review), and may be useful also in the area of service learning.

Obtaining balance on important confounding factors may be difficult when students are not randomised or a natural experiment is not available, which adds to the importance of statistically controlling for relevant factors. In this review, we would have judged the risk of bias due to confounding to be of less concern had the primary study authors controlled for more relevant factors in their analyses. As data on for example performance at baseline, grade level, gender, or socioeconomic background were available in some studies judged to be at Critical risk of bias, we would recommend that this information is also used in the analyses to control for important confounding factors.

Lastly, in calling for more randomised trials and quasi‐experimental studies, we do not want to downplay the importance of qualitative methods. On the contrary, qualitative methods are likely necessary to learn more about how effects come about and why they might differ between contexts. That is, a high‐quality randomised trial ought to be combined with a detailed qualitative investigation of for example implementation fidelity and the operationalisation of service learning across sites.

## DATA AND ANALYSES

**1 Academic success**

**Table:** 

**Outcome or subgroup**	**Studies**	**Participants**	**Statistical method**	**Effect estimate**
1.1 Grade point average	3		Std. Mean Difference (IV, Random, 95% CI)	0.09 [−0.02, 0.21]
1.2 Reading	2		Std. Mean Difference (IV, Random, 95% CI)	0.04 [−0.08, 0.16]
1.3 Math	2		Std. Mean Difference (IV, Random, 95% CI)	0.21 [0.09, 0.33]
1.4 Absences	2		Std. Mean Difference (IV, Random, 95% CI)	0.03 [−0.10, 0.16]

**2 Personal and social skills**

**Table:** 

**Outcome or subgroup**	**Studies**	**Participants**	**Statistical method**	**Effect estimate**
2.1 Self‐esteem	2		Std. Mean Difference (IV, Random, 95% CI)	0.13 [−0.14, 0.40]
2.2 Locus of control	3		Std. Mean Difference (IV, Random, 95% CI)	0.07 [−0.04, 0.18]

**3 Risk behaviour**

**Table:** 

**Outcome or subgroup**	**Studies**	**Participants**	**Statistical method**	**Effect estimate**
3.1 Ever been/made someone pregnant	2		Odds Ratio (IV, Random, 95% CI)	1.05 [0.63, 1.74]
3.2 Risky sex	3		Odds Ratio (IV, Random, 95% CI)	0.96 [0.74, 1.25]
3.4 Ever been/made someone pregnant Follow up	2		Odds Ratio (IV, Random, 95% CI)	0.84 [0.39, 1.82]
3.5 Risky sex Follow up	3		Odds Ratio (IV, Random, 95% CI)	0.89 [0.73, 1.10]

**4 Sensitivity**

**Table:** 

**Outcome or subgroup**	**Studies**	**Participants**	**Statistical method**	**Effect estimate**
4.2 Sensitivity Grade point average	3		Std. Mean Difference (IV, Random, 95% CI)	0.10 [−0.02, 0.22]
4.5 Sensitivity Reading	2		Std. Mean Difference (IV, Random, 95% CI)	0.05 [−0.08, 0.18]
4.6 Sensitivity Math	2		Std. Mean Difference (IV, Random, 95% CI)	0.21 [0.08, 0.34]
4.7 Sensitivity Self‐esteem	2		Std. Mean Difference (IV, Random, 95% CI)	0.09 [−0.27, 0.46]

## AUTHOR CONTRIBUTIONS

*Content*: Trine Filges, Jens Dietrichson and Nina T. Dalgaard. *Systematic review methods*: Trine Filges, Jens Dietrichson and Nina T. Dalgaard. *Statistical analysis*: Trine Filges and Jens Dietrichson. *Information retrieval*: Bjørn Viinholt.

## CONFLICT OF INTERESTS

The authors declare that there are no conflict of interests.

## SOURCES OF SUPPORT

### 1 Internal sources

• VIVE Campbell, Denmark

## Supporting information

Supporting information.

Supporting information.
