# Supplementary material for: Service learning for improving academic success in students in grade K to 12: A systematic review
Source: Campbell Syst Rev. 2022 Jan 7;18(1):e1210. doi: 10.1002/cl2.1210 (PMC8741202; doi:10.1002/cl2.1210)
Supplement: Supplementary file 1 — Supporting information. [file CL2-18-e1210-s002.docx]

# Appendices

## 1 First and second level screening

First level screening is on the basis of titles and abstracts.  Second level is on the basis of full text

Reference  id. No. :

Reviewers initials:

Source:

Year of publication:

Country/countries of origin:

Author(s):

The study will be excluded if one or more of the answers to question 1-3 are ‘No’. If the answers to question 1 to 3 are ‘Yes’ or ‘Uncertain’, then the full text of the study will be retrieved for second level eligibility. All unanswered questions need to be posed again on the basis of the full text. If not enough information is available, or if the study is unclear, the author of the study will be contacted if possible.

**Screening questions:**

1.       Does the study focus on service learning?

Yes - include

No – if no then stop here and exclude

Uncertain - include

Question 1 guidance:

The intervention in this review is service learning. Studies considering extra-curriculum activities or stand-alone volunteer or community activity will not be eligible.

2.      Are the participants children in grades kindergarten to 12 (or the equivalent in European countries) in general education?

Yes - include

No – if no then stop here and exclude

Uncertain - include

Question 2 guidance:

Regular private, public or boarding schools are eligible. We exclude children in home-school. In some countries, kindergarten can however refer to preschool programs outside of primary school and include ages down to 2 years. Service learning targeting such populations will be excluded; that is, kindergarten must be considered a part of primary school for a study to be included.

3.      Is the report/article a quantitative evaluation study with a comparison condition?

Yes - include

No – if no then stop here and exclude

Uncertain - include

Question 3 guidance:

We are only interested in primary quantitative studies with a comparison group, where the authors have analysed the data. We are not interested in theoretical papers on the topic or surveys/reviews of studies of the topic. (This question may be difficult to answer on the base of titles and abstracts alone.)

## 2 Data extraction

| **Names of author(s)** |
| --- |
| **Title** |
| **Language** |
| **Journal** |
| **Year** |
| **Country** |
| **Type of school – private, boarding, public** |
| **Participant characteristic (age, grade level, gender, socioeconomic status, ethnicity )** |
| **Programme feature L***inking to curriculum (answer yes or no)*, a yes requires that the study as a minimum have reported, having clear goals for the programme that align with the curriculum, and containing corresponding activities to match those goals. |
| **Programme feature** *Youth voice,* code yes when it is reported that students were involved in the  planning, implementation, or evaluation process of the program |
| **Programme feature** *Community involvement,* code yes if it is reported that the community has some part in the programme besides providing a place for students to serve |
| **Programme feature R***eflection*, code yes if some type of reflection activity (e.g., using journals, having discussions in class or in small groups, writing essays about the service experience, presenting to the class what was learned, or reflecting individually with the teacher or site supervisor are reported |
| **Programme feature** *Duration* **(**number of weeks, one semester, one school year**)** |
| **Programme feature** *Intensity* **(**number of community service hours per week/month**)** |
| **Type of data used in study (administrative, questionnaire, other (specify))** |
| **Level of aggregation (individual or class)** |
| **Time period covered by analysis (divide into intervention and follow up)** |
| **Sample size (divide into treated/comparison)** |

## 3 User guide for unobservables

Systematic baseline differences between groups can compromise comparability between groups. Baseline differences can be observable (e.g. age and gender) and unobservable (to the researcher; e.g. motivation and ‘ability’). There is no single non-randomised study design that always solves the selection problem. Different designs solve the selection problem under different assumptions and require different types of data. Especially how different designs deal with selection on unobservables varies. The “right” method depends on the model generating participation, i.e. assumptions about the nature of the process by which participants are selected into a programme.

As there is no universal correct way to construct counterfactuals we will assess the extent to which the identifying assumptions (the assumption that makes it possible to identify the counterfactual) are explained and discussed (preferably the authors should make an effort to justify their choice of method).  We will look for evidence that authors using e.g. (this is NOT an exhaustive list):

**Natural experiments:**

Discuss whether they face a truly random allocation of participants and that there is no change of behaviour in anticipation of e.g. policy rules.

**Matching (including propensity scores):**

Explain and discuss the assumption that there is no selection on unobservables, only selection on observables.

**(Multivariate, multiple) Regression:**

Explain and discuss the assumption that there is no selection on unobservables, only selection on observables. Further discuss the extent to which they compare comparable people.

**Regression Discontinuity (RD):**

Explain and discuss the assumption that there is a (strict!) RD treatment rule. It must not be changeable by the agent in an effort to obtain or avoid treatment. Continuity in the expected impact at the discontinuity is required.

**Difference-in-difference (Treatment-control-before-after):**

Explain and discuss the assumption that the trends in treatment and control groups would have been parallel, had the treatment not occurred.

## 4 Justification of exclusion of studies using an instrumental variable (IV) approach

Studies using instrument variables (IV) for causal inference in non-randomised studies will not be included as the interpretation of IV estimates is challenging. IV only provides an estimate for a specific group namely, people whose behaviour change due to changes in the particular instrument used. It is not informative about effects on never-takers and always-takers because the instrument does not affect their treatment status. The estimated effect is thus applicable only to the subpopulation whose treatment status is affected by the instrument. As a consequence, the effects differ for different IVs and care has to be taken as to whether they provide useful information. The effect is interesting when the instrument it is based on is interesting in the sense that it corresponds to a policy instrument of interest. Further, if those that are affected by the instrument are not affected in the same way the IV estimate is an average of the impacts of changing treatment status in both directions, and cannot be interpreted as a treatment effect. To turn the IV estimate into a LATE requires a monotonicity assumption. The movements induced by the instrument go in one direction only, from no treatment to treatment. The IV estimate, interpreted as a LATE, is only applicable to the complier population, those that are affected by the instrument in the ‘right way’. It is not possible to characterise the complier population as an observation’s subpopulation cannot be determined and defiers do not exist by assumption.

In the binary-treatment–binary-instrument context, the IV estimate can, given monotonicity, be interpreted as a LATE; i.e. the average treatment effect for the subpopulation of compliers. If treatment or instruments are not binary, interpretation becomes more complicated. In the binary-treatment–multivalued-instrument (ordered to take values from 0 to *J*) context, the IV estimate, given monotonicity, is a weighted average of pairwise LATE parameters (comparing subgroup *j* with subgroup *j*−1). The IV estimate can thus be interpreted as the weighted average of average treatment effects in each of the *J* subgroups of compliers. In the multivalued-treatment (ordered to take values from 0 to *T*) – multivalued-instrument (ordered to take values from 0 to *J*) context, the IV estimate for *each pair of instrument values*, given monotonicity, is a weighted average of the effects from going from *t*-1 to *t* for persons induced by the change in the value of the instrument to move from any level below *t* to the level *t* or any level above. Persons can be counted multiple times in forming the weights.
